# Generation of biologically responsive colon-like intestinal tissue patches from human induced pluripotent stem cells using a rapid co-differentiation platform

**DOI:** 10.1186/s13287-026-05006-4

**Published:** 2026-04-09

**Authors:** William Dalleywater, Alexander V. Predeus, Batuhan Cakir, Pavel Mazin, Jayakumar Vadakekolathu, Sergio Rutella, Marian L. Meakin, Alison A. Ritchie, Shamir Montazid, Sara Cuevas Ocaña, Nadine Holmes, Victoria Wright, Fei Sang, Silvia Santoni, Adam Bills, Declan Sculthorpe, Rasa Elmentaite, Sarah A. Teichmann, Shazia Irshad, Ian Tomlinson, Andrew Silver, Ricky D. Wildman, Nicholas R. F. Hannan, Felicity R. A. J. Rose, Mohammad Ilyas

**Affiliations:** 1 Unit of Translational Medical Sciences, School of Medicine, Biodiscovery Institute, University of Nottingham, Nottingham, UK; 2 Department of Cellular Pathology, Queen’s Medical Centre, Nottingham University Hospitals NHS Trust, Nottingham, UK; 3 Wellcome Sanger Institute, Hinxton, Cambridge, UK; 4 John van Geest Cancer Research Centre, Nottingham Trent University, Nottingham, UK; 5 Nuffield Division of Clinical Laboratory Sciences, Radcliffe Department of Medicine, Oxford, UK; 6 Department of Oncology, University of Oxford, Oxford, UK; 7 School of Life Sciences, University of Nottingham, Nottingham, UK; 8 School of Pharmacy, Biodiscovery Institute, University of Nottingham, Nottingham, UK; 9 Cambridge Stem Cell Institute, University of Cambridge, Cambridge, UK; 10 Centre for Genomics and Child Health, Blizard Institute, Barts and The London School of Medicine and Dentistry, Queen Mary University of London, London, UK; 11 Faculty of Engineering, University of Nottingham, Nottingham, UK

**Keywords:** Induced pluripotent stem cells, Intestine, Colon, Co-differentiation, Tissue engineering

## Abstract

The intestinal mucosa is a complex functional layer which is formed from a diverse range of cell types that include epithelial cells (within crypts and villi) and an array of mesenchymal cells. Many intestinal diseases involve loss of the surface mucosa which can be difficult to restore, and which delays healing and return to normal function. We reason that development of a transplantable intestinal mucosal tissue graft may be a potential therapeutic strategy to aid healing. To be clinically useful, such a tissue graft would need to be capable of rapid production, avoid the risk of host rejection and be demonstrably safe. To create a potential intestinal graft, we developed a novel early-stage human induced pluripotent stem cell (hiPSC) co-differentiation platform capable of generating multiple intestinal cell lineages (epithelial, mesenchymal and endothelial) in 8 days. This protocol is simple to implement, serum-free and greatly reduces the use of animal products. We confirmed the identity of cells by demonstrating that these cells had RNA and protein expression profiles typical of intestinal cell lineages. In particular, we used bulk and single-cell RNA sequencing to characterise global cellular transcriptional profiles robustly and showed that the cells have intestinal identity with early polarisation towards colonic differentiation. The results were replicated across multiple hiPSC lines and in an independent centre. We further cultured the derived cells on collagen hydrogels to form colon-like intestinal patches (CL-IPs). When transplanted into mouse subcutis, CL-IPs formed into colon-like tissue structures, including crypts, stromal and muscle layers. They also developed human-origin vasculature which underwent anastomosis with the murine vasculature to transport murine blood into the graft. Teratoma assays and molecular analyses showed no evidence of residual pluripotency. While at an early stage, this platform shows great potential for further development as a potential source for novel intestinal mucosal regeneration therapy. In addition, the platform is physiologically relevant and thus shows promise as the basis for a new generation of in vitro models of intestinal pathobiology.

**Graphical abstract:**

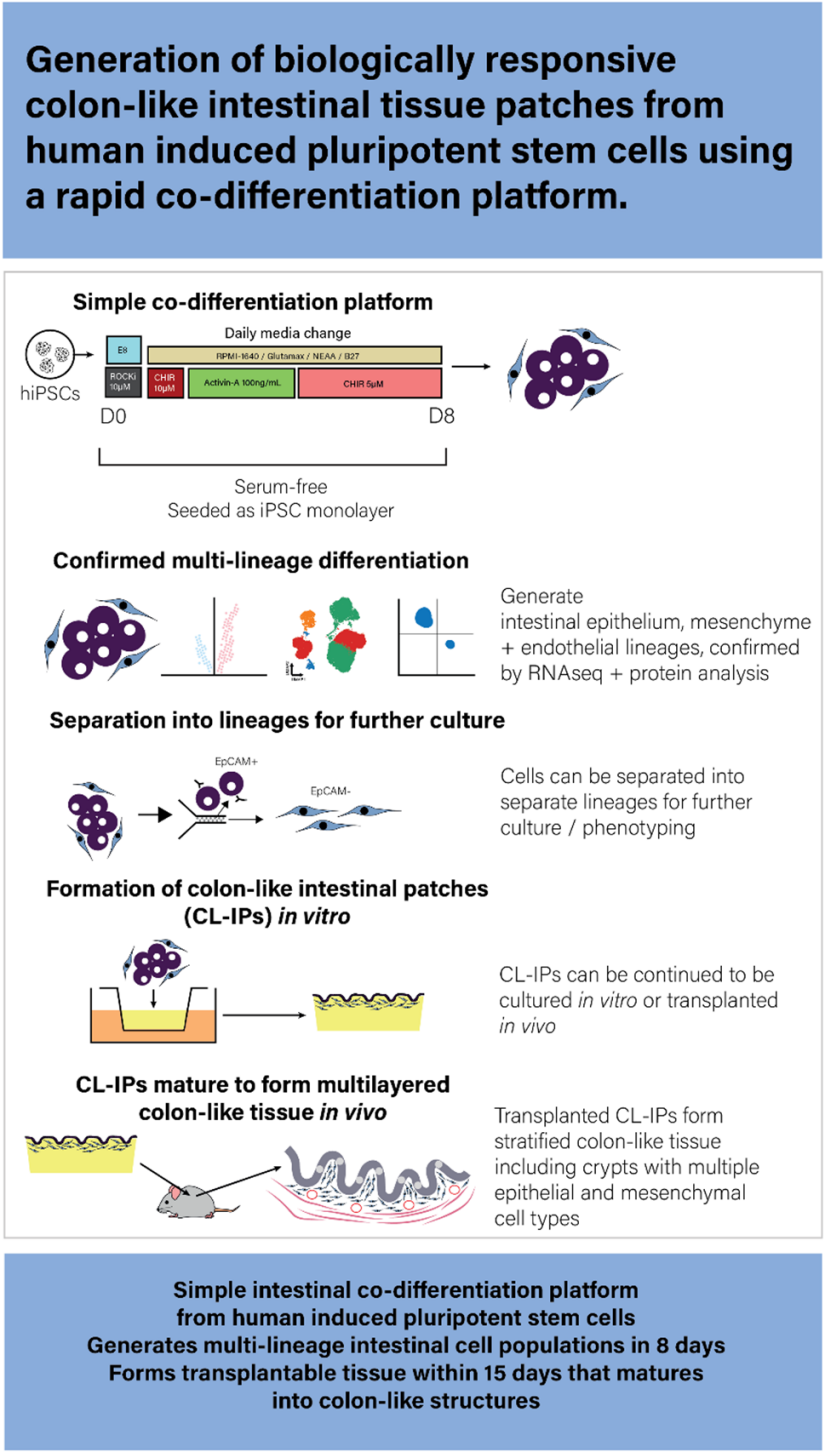

**Supplementary Information:**

The online version contains supplementary material available at 10.1186/s13287-026-05006-4.

## Introduction

Intestinal diseases are common and constitute a major health burden [1]. Many such diseases involve loss of the surface mucosa. As the mucosa is the main functional layer of the intestine – involved in absorption of nutrients and water – dysfunction results in many of the symptoms of bowel disease, such as diarrhoea, dehydration, nutrient deficiencies and haemorrhage. Loss of mucosal integrity is a prominent feature in inflammatory bowel disease (IBD) and thus restoring healthy mucosa may aid in breaking the cycle of chronic inflammation and microbial colonisation, to aid healing [2–4]. The mucosa is thus a focus for regeneration therapies [3, 5].

The intestinal mucosa is characterised by epithelial crypts supported by a range of mesenchymal cells including fibroblasts and endothelial cells and a deep band of smooth muscle, called *muscularis mucosae* [6–8]. These cell populations derive from endoderm and splanchnic mesoderm during embryogenesis and arise in tandem from the developing gut tube. Other cell populations, such as haemopoietic and neural cells migrate into the intestinal mucosa during embryonic life from other germ layers [8]. Interactions between epithelial and neighbouring stromal cells are particularly important in maintaining normal intestine mucosal tissue homeostasis, through the establishment of signalling morphogen gradients. These factors promote appropriate cell differentiation to allow normal gut function (for example, cells such as goblet cells, absorptive cells and endocrine cells) [9]. They also create a stem cell niche that facilitates replacement of differentiated cells that are shed from the crypt apex, while shielding stem cells from toxicity inherent to the gut luminal contents [10].

Since intestinal pathologies often involve the breakdown of these complex interactions between epithelial and mesenchymal populations [7], any cell supply source for intestinal regeneration or modelling should be capable of generating all these cell types. The most popular existing intestinal cells for disease modelling are in vitro organoids and recent improvements in methodology have increased their utility. However, these models require access to primary tissue (usually surgical resection specimens) and are not able to replicate fully the complex cellular population profile and 3D intestinal architecture [11, 12]. They are slow to expand and may not yield enough cells to be used therapeutically. Furthermore, for complex tissue regeneration or modelling, each cell lineage must be isolated and expanded separately and then optimised in cell culture [12, 13].

Our aim was to generate human intestinal cell populations from hiPSCs and illustrate their potential to reconstruct normal intestinal mucosal tissue in vitro and in vivo. For successful in vivo engraftment, we reasoned that a transplant should resemble, as much as possible, normal intestine in organisation and cell types (an epithelium with underlying stroma). We hypothesised that a protocol with reproducible co-differentiation of hiPSCs into various cell populations present in normal colonic mucosa would represent a more developmentally relevant approach, given the importance of inter-lineage cues in shaping differentiated cell phenotypes [6, 14–16]. These would then need to be grown on an in vitro scaffold allowing assembly into architecturally normal mucosa, and which would be robust enough to allow transplantation.

Here, we present a rapid and robust co-differentiation protocol for generating intestinal cells from hiPSCs that can develop into intestinal mucosal tissue, with molecular and histological features recapitulating colonic cell populations.

## Results

### Defining a novel serum-free protocol

**Fig. 1:**
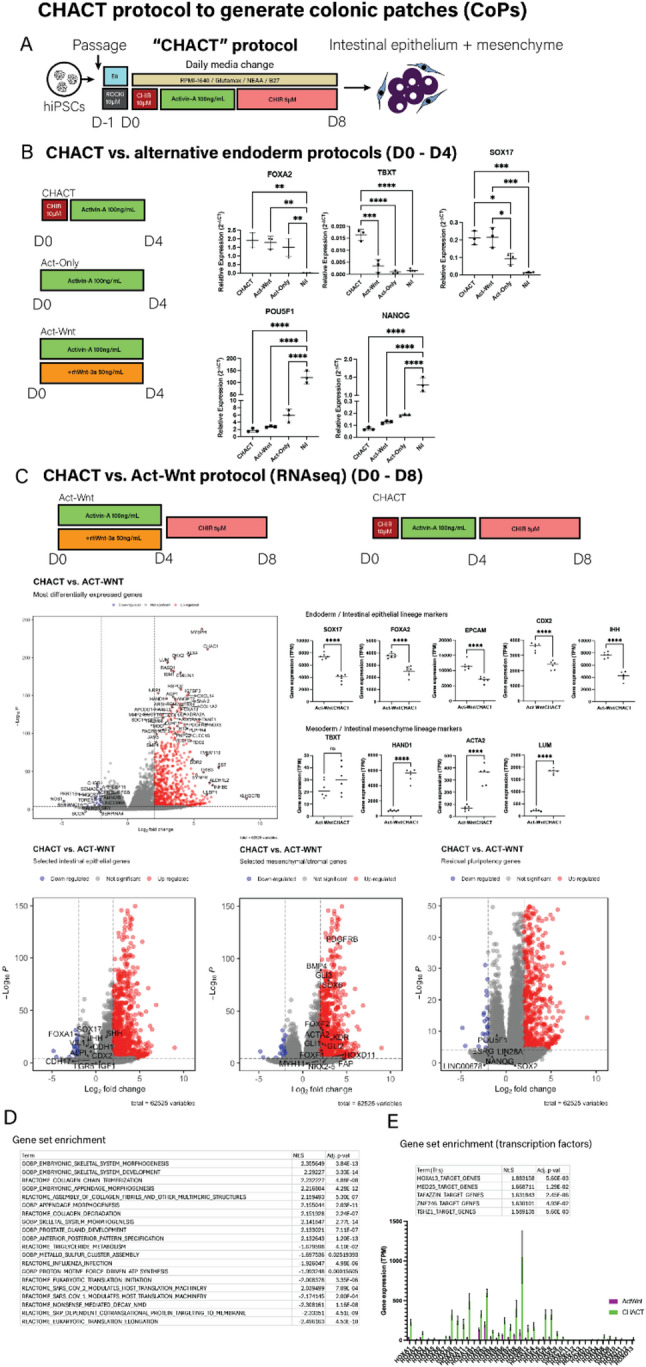
An 8-day protocol capable of deriving multiple intestinal cell populations. **A** Overview of the novel differentiation protocol (CHIR-Activin protocol–CHACT, see also Table 1). **B** Comparison of CHACT protocol with alternative protocols (see also Table 1). Expression of endoderm (*FOXA2*,* SOX17*)/mesoderm (*TBXT/Brachyury*) and pluripotency (*POU5F1/NANOG*) markers by qRT-PCR at day 4 of each protocol demonstrates enhanced co-differentiation with CHACT (see methods) (*n* = 3 independent experiments). Error bars indicate standard deviation. Statistical significance assessed by one-way ANOVA. **C** CHACT results in enrichment of mesenchyme-related genes (e.g. *HAND1*,* LUM*,* PDGFRB*,* TNNT1*) [5, 6] compared with Act-Wnt protocol [24]. Cells were differentiated according to the protocols shown and then compared by mRNA-sequencing (*n* = 6). Volcano plots illustrate top differentially expressed genes and specific epithelial and mesenchymal subsets. Lineage markers for endoderm/mesoderm and intestinal epithelial and mesenchymal cell populations were assessed between Act-Wnt and CHACT conditions (ns = not significant; **** = *p* < 0.0001). **D** Gene set enrichment analysis (GO-Biological Process/Reactome) between Act-Wnt and CHACT differentiated cells at day 8, normalised enrichment score (NES; positive = CHACT enriched/negative = Act-Wnt enriched) and adjusted p-value shown for each term (top 10 up- and down-regulated displayed), showing enrichment of mesenchyme-related terms in cells after CHACT differentiation. **E** Gene set enrichment analysis of transcription factor targets (msigdb: TFT-GTRD) demonstrated increased activity of HOXA13 after CHACT differentiation. TPM values for HOX genes are shown and demonstrated enrichment of posterior HOX related genes after CHACT

To replicate the complexity of the mucosa, we aimed to co-differentiate hiPSCs simultaneously to form both endoderm and mesoderm. We favoured a co-differentiation approach as this would allow interactions between different cell populations during differentiation, mimicking early developmental events in gut tube and early intestinal development. Many published protocols (outlined in Suppl. Table 1) rely on poorly defined media components which are prone to batch-to-batch variation (such as conditioned media and animal derived serum), use antibiotics or require complex and lengthy culture conditions [17–22]. As our aim was to design a protocol that could be suitable for eventual transplantation and for easy adoption in other laboratories, we reasoned that using serum-containing components would limit application [18]. Thus, we set out to explore protocol options in which differentiation could be achieved with recombinant or small molecules only. The resulting co-differentiation protocol replaced serum with defined products, reduced recombinant proteins and was conducted in antibiotic-free conditions. This protocol (Fig. 1A; Table 1, CHIR-99021-ACTivin A: CHACT protocol) uses RPMI-1640 media supplemented with B27 and non-essential amino acids and comprises 3 main steps: hiPSCs are stimulated for 24 h with CHIR-99021 (10 µM); differentiating hiPSCs cells are cultured with recombinant Activin-A (100 ng mL^− 1^) for 3 days and the last four days of culture with a lower concentration of CHIR-99021 (5 µM). CHIR-99,021 is a small molecule inhibitor of GSK-3β (part of the β-catenin destruction complex), through which it activates the canonical Wnt pathway. We tested this novel protocol (CHACT protocol) against two alternatives that contain the major non-serum components utilised in other widely used published protocols [18, 23] - Activin (Act)-Wnt and Act-Only (Table 1; Fig. 1B).

**Table 1:** Summary of differentiation protocols tested

Timepoint	CHACT protocol	Act-Wnt protocol (23)	Act-only protocol (18)
Day − 1	hiPSCs seeded onto Matrigel^®^ coated plate		
Day 0	CHIR-99021 (10 µM)	Activin A (100 ng mL^− 1^) + Wnt-3a (25 ng mL^− 1^)	Activin A (100 ng mL^− 1^)
Day 1	Activin A (100 ng mL^− 1^)	Activin A + Wnt-3a	Activin A
Day 2	Activin A	Activin A + Wnt-3a	Activin A
Day 3	Activin A	Activin A + Wnt-3a	Activin A
Day 4	CHIR-99021 (5 µM)	CHIR-99021 (5 µM)	CHIR-99021 (5 µM)
Day 5	CHIR-99021 (5 µM)	CHIR-99021 (5 µM)	CHIR-99021 (5 µM)
Day 6	CHIR-99021 (5 µM)	CHIR-99021 (5 µM)	CHIR-99021 (5 µM)
Day 7	CHIR-99021 (5 µM)	CHIR-99021 (5 µM)	CHIR-99021 (5 µM)
Day 8	End of protocol		

Baseline media = RPMI-1640 with glutamax + B27 supplement (1X) + non-essential amino acids (1X). Media changed daily during all differentiation protocols. Protocol abbreviations: *CH*IR-99021 + *Act*ivin A: CHACT protocol, Activin A + Wnt-3a: Act-Wnt protocol, Activin A–Act-Only protocol

### CHACT protocol leads to co-differentiation of hiPSCs into endoderm and mesoderm

By day 4 of the CHACT protocol, there was up-regulation of endoderm markers *SOX17* and *FOXA2* and the mesoderm marker, *TBXT* (Brachyury) which was not seen in comparative protocols (Fig. 1B) [23]. Act-Wnt and CHACT protocols showed similar levels of *SOX17*, in both conditions significantly higher than Act-only. All protocols showed a strong downregulation of pluripotency markers, *POU5F1* and *NANOG*. From this initial characterisation, Act-Wnt/CHACT showed similar induction of endoderm markers while CHACT showed evidence of mesoderm induction, we therefore reasoned that these protocols would be most promising to test further.

We compared transcriptomic profiles by RNA-Seq at Day 8 in cells cultured using the CHACT protocol with cells generated using the Act-Wnt protocol (Fig. 1C; Table 1) [18, 19]. Differentiated cells derived using both protocols showed similar levels of expression of intestinal epithelial markers (Fig. 1C, lower left panel), but cells grown under CHACT were also strongly enriched for stroma-related genes including *HAND1*,* LUM*,* BMP4*,* ACTA2*,* SOX6* and *KDR* (Fig. 1C, lower right panel) [6, 7]. Gene set enrichment analysis (Fig. 1D) further demonstrated enrichment of mesenchyme related terms in cell populations generated using the CHACT protocol. In addition, analysis of transcription factors using gene set enrichment analysis showed increased activity of *HOXA13* (Fig. 1E). Analysis of HOX gene expression showed particularly high expression of posterior HOX genes including *HOXA10*,* HOXA11*,* HOXA13* and *HOXB9* after the CHACT protocol [17, 24, 25].

**Fig. 2:**
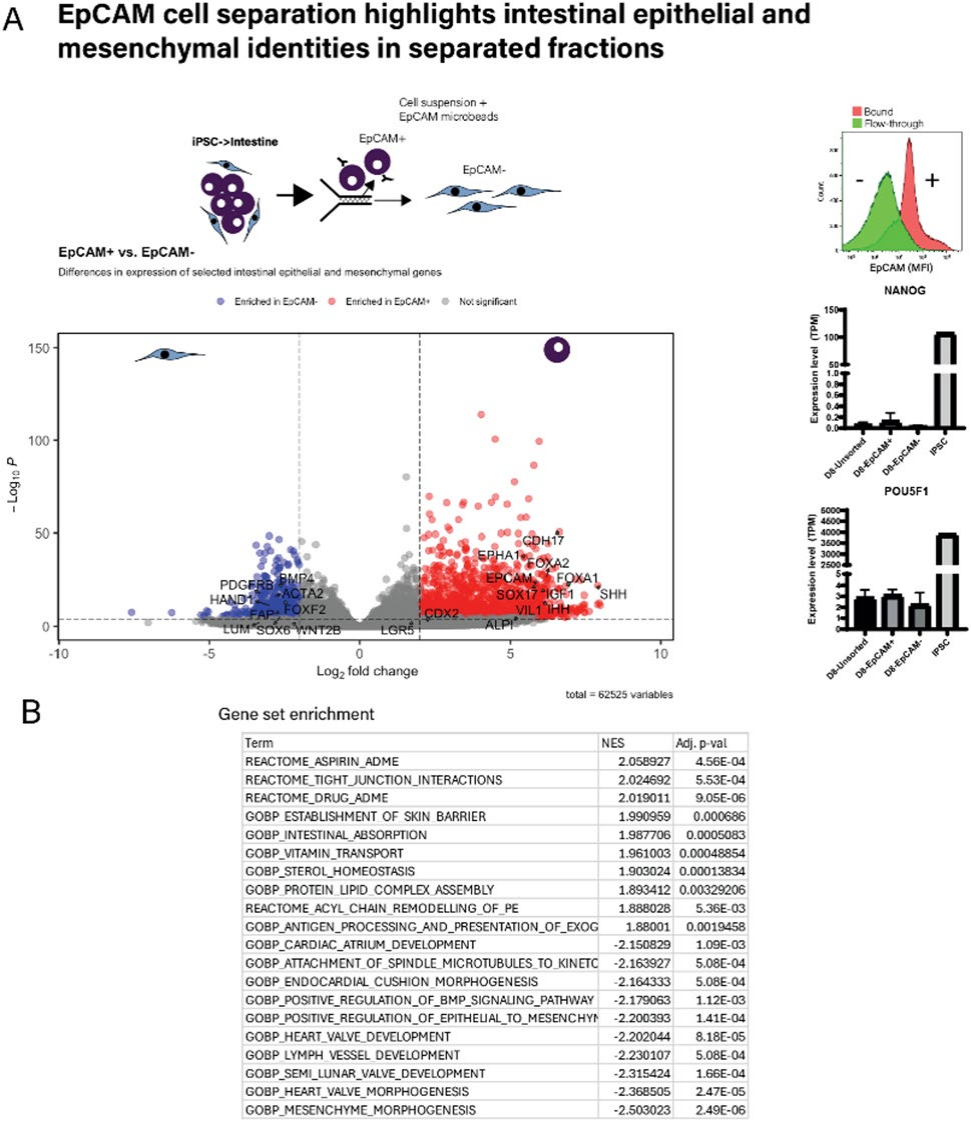
Separating cells based on EpCAM expression demonstrated intestinal epithelial and mesenchymal identities. **A** CHACT co-differentiated cells when MACS separated using EpCAM expression showed enrichment of intestinal epithelial genes in the EpCAM+ fraction (e.g. *CDH17*,* EPCAM*,* VIL1*,* ALPI*) and intestinal mesenchymal genes in the EpCAM- fraction (e.g. *ACTA2*,* BMP4*,* SOX6*,* LUM*). Cells were differentiated and then separated based on EpCAM expression (MACS) and profiled by flow cytometry/mRNA-sequencing. Representative flow cytometry plot following separation by MACS^®^ cell separation of separated populations (*n* = 3). Volcano plots demonstrate top genes enriched in EpCAM + and EpCAM- populations. **B** Gene set enrichment analysis (GO-Biological Process/Reactome) in EPCAM + and EPCAM- separated cell populations after CHACT protocol highlighted enrichment of epithelial-related terms, including terms related to intestinal epithelium, in the EPCAM+ population and mesenchyme and extracellular matrix related terms in the EPCAM- population

We further validated cell identity by separating cultures at the end of the CHACT protocol into enriched “epithelial” and “mesenchymal” groups using magnetic cell separation (MACS) based on EpCAM expression (a marker of epithelium) (Fig. 2A). Expression profiling by mRNA-Seq confirmed intestinal epithelial identity of the EpCAM+ group (compared with the EpCAM- group) including enrichment of general epithelial markers (*SOX17*,* LGR5*,* SHH*,* IHH)* and specific intestinal markers (*CDH17*,* ALPI*,* VIL1*). In contrast to the EpCAM+ group, the EpCAM- group showed enrichment for mesenchymal markers including *ACTA2*,* BMP4*,* LUM* and *SOX6* [6, 7] (Fig. 2A). There was no difference in pluripotency marker (SOX2, OCT4 etc.) expression between the separated groups (Fig. 2A). Moreover, expression of these was > 1000 fold lower in differentiated cells compared with undifferentiated hiPSCs. Gene set enrichment analysis showed enrichment of intestinal epithelial related terms in the EPCAM+ population and mesenchyme and vasculature related terms in the EPCAM- population (Fig. 2B). Our data were replicated and were consistent among four different hiPSC lines (REBLPAT: male, dermal fibroblast; BE31: female, dermal fibroblast; BE32: female, dermal fibroblast; OSTI GI4380: male, intestinal fibroblast; Fig.S1A and B). The protocol was performed independently by collaborators in another academic institution (Fig.S1B) and yielded identical results thereby demonstrating the robustness and the ease of implementing the protocol. In addition, EpCAM+ cells showed abundant *LGR5* transcripts by RNAscope, and formed proliferating (Ki67+) organoids when encapsulated in Matrigel. Finally, we applied the protocol to a commercial healthy control iPSC line (SCTi-003a: female, peripheral T-cell) (Fig. S1C) and showed consistent expression of markers of intestinal epithelial (flow cytometry – EPCAM / qRT-PCR: *CDX-2 / MUC2*), endothelial (flow cytometry – VE-cad) and mesenchymal (qRT-PCR: *ACTA2 / SOX6*) differentiation between our donor (REBLPAT) and commercial line. We have shown that our protocol is capable of intestinal epithelial and mesenchymal differentiation using five iPSC lines and demonstrate expected epithelial differentiation within the range of 70–80% across these lines.

### Single-cell RNA sequencing confirms co-differentiation of hiPSCs into multiple epithelial and mesenchymal sub-populations

**Fig. 3:**
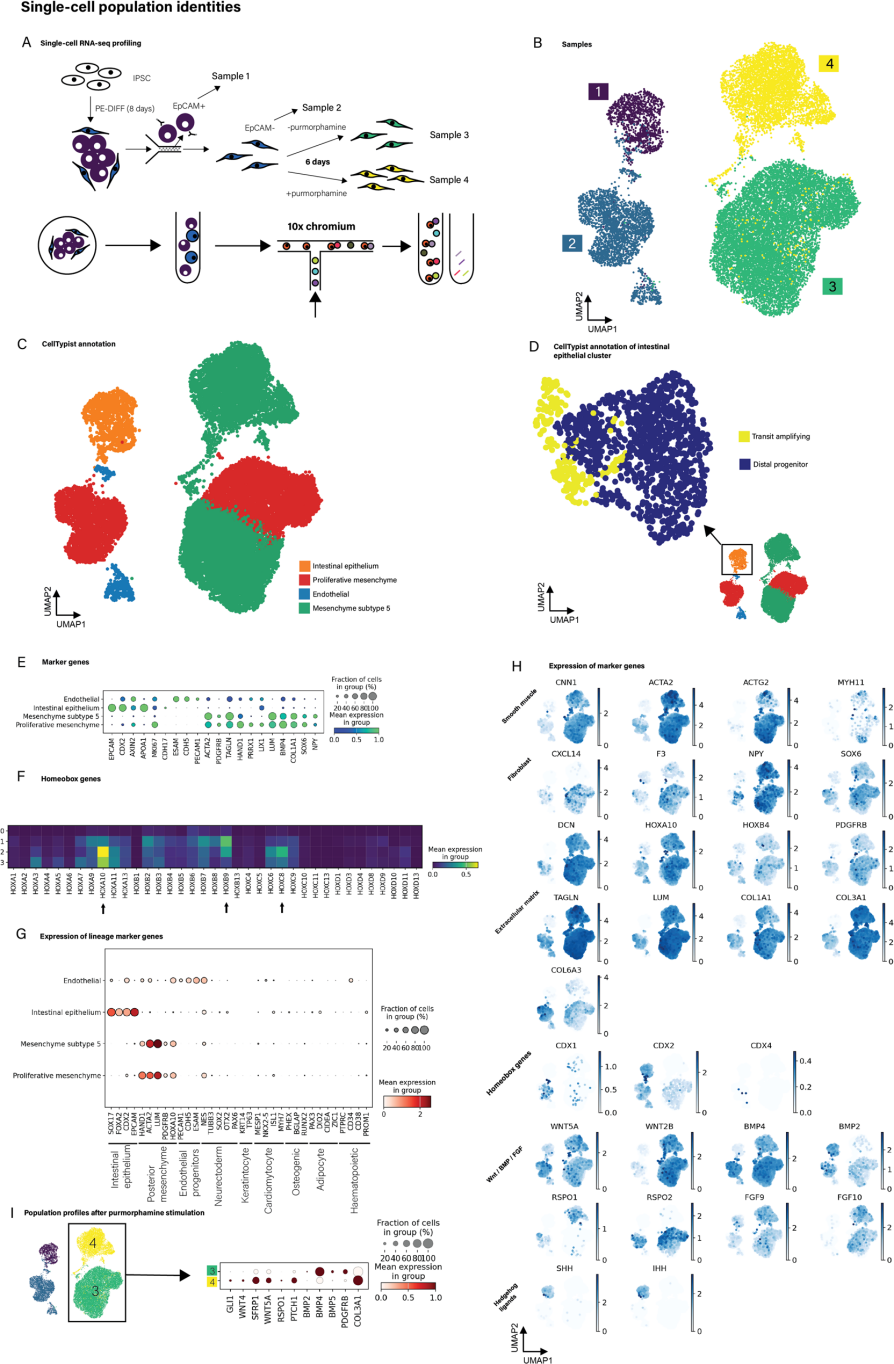
Single-cell RNAseq of separated cells after the CHACT protocol demonstrates intestinal epithelial, mesenchymal and endothelial population identities with expression of marker genes recapitulating intestinal population heterogeneity. **A** Overview of study design; sample 1: day 8, EpCAM+; sample 2: day 8, EpCAM-; sample 3: day 14 EpCAM- in monoculture after separation; sample 4: day 14 EpCAM- (monoculture) with purmorphamine treatment. **B** Uniform manifold approximation and projection (UMAP) leiden clustering of all samples. **C** Automated annotation using the CellTypist algorithm of each cell population according to the “Developing Human Organs” model, showing intestinal epithelial, endothelial and mesenchymal identities. **D** Automated annotation using the CellTypist algorithm of the intestinal epithelial population according to the “Cells Intestinal Tract” model, showing distal progenitor and transit amplifying identities. **E** Dotplot of top marker genes of each population grouped by CellTypist (Developing Human Organs) annotations. **F** Mesenchyme (samples 1, 2, 3) are enriched for posterior homeobox genes. Heatmap showing expression of Hox genes in each sample, arrows indicate posterior Hox genes. **G** Expression of markers of lineage identities as indicated were assessed across the four main populations derived using CHACT. These demonstrated enrichment of intestinal epithelial, mesenchymal and endothelial progenitors, but no specific enrichment of alternative lineages. **H** Feature plots of marker genes corresponding to smooth muscle and fibroblast lineages, Wnt/BMP/FGF and Hedgehog signalling pathways and extracellular matrix. **I** Comparison of mesenchyme with (sample 4) and without (sample 3) purmorphamine treatment to generate GLI1^+^/crypt niche phenotype, with a dotplot of key signalling and marker genes in each of the treated (sample 4) and control (sample 3) samples

To characterise the cellular populations in the epithelial and mesenchymal compartments, sc-RNAseq was performed (i) following cell sorting (EpCAM + and EpCAM- cells) immediately at the end of CHACT protocol on day 8 and (ii) following a further 6 days of in vitro monoculture of EpCAM- cells (Fig. 3A/B). For the latter, from day 8 to day 14, EpCAM- cells were cultured with or without purmorphamine to study differentiation of the stromal population in more detail. Single-cell expression profiles of cells were compared to publicly available single-cell reference atlases generated as part of the human cell atlas project, using CellTypist (“Developing_Human_Organs”) (Fig. 3C) [2, 6, 26]. This demonstrated a diversity of emerging intestinal epithelial and stromal populations indicative of mesoderm differentiation towards the cell types seen in the mature intestinal stroma [25, 26]. The epithelial cell population was separated into subsets from the main dataset and mapped to the human gut cell atlas (“Cells_Intestinal_Tract”); revealing that epithelial cells mapped predominantly to “distal progenitors” as well as “transit amplifying” cells, supporting colonic identity (Fig. 3D). The identity of all populations was further confirmed by demonstrating enrichment of marker genes for intestinal epithelium, endothelium, mesoderm and more mature mesenchyme (Fig. 3E). Furthermore, we demonstrated a posterior homeobox programme (important for specifying the fate of the mesoderm towards an appropriate intestinal mesenchyme), dominated by *HOXA10/11*,* HOXB9* and *HOXC6-9* (Fig. 3F). Our data showed expression of *CDX2* in both epithelial and mesenchymal populations at day 8, which is consistent with a recent finding that *CDX2*, although ultimately expressed only in mature epithelial tissue, is required for both intestinal endoderm and mesoderm fate specification [25] (Fig. 3G). Within later mesenchymal clusters (clusters annotated 3 + 4, Fig. 3B/G) subclusters of cells showed enrichment for expression of smooth muscle markers (*MYH11*,*ACTA2*,*CNN1*,*ACTG2*) whilst others demonstrated a phenotype typical of specific mature fibroblast subsets (*CXCL14*,*F3*,*RSPO2*,*NPY*) [6]. The Hedgehog pathway is important for directing intestinal stromal lineages, as demonstrated in earlier experiments where stromal cells were stimulated with purmorphamine. We found that Hedgehog ligands were restricted to the epithelial population (Fig. 3G). To understand further the effects on population profiles, stromal cells were profiled by single-cell RNAseq after treating with purmorphamine (Fig. 3H). This caused enrichment of *GLI1* and *PTCH1* thereby confirming a Hedgehog response. Upregulation of *WNT4* and *SFRP1* and downregulation of BMP ligands were observed; features that recapitulate the phenotype of WNT-secreting crypt-niche mesenchyme described in mice [27].

### Polarisation towards colonic identity

**Fig. 4:**
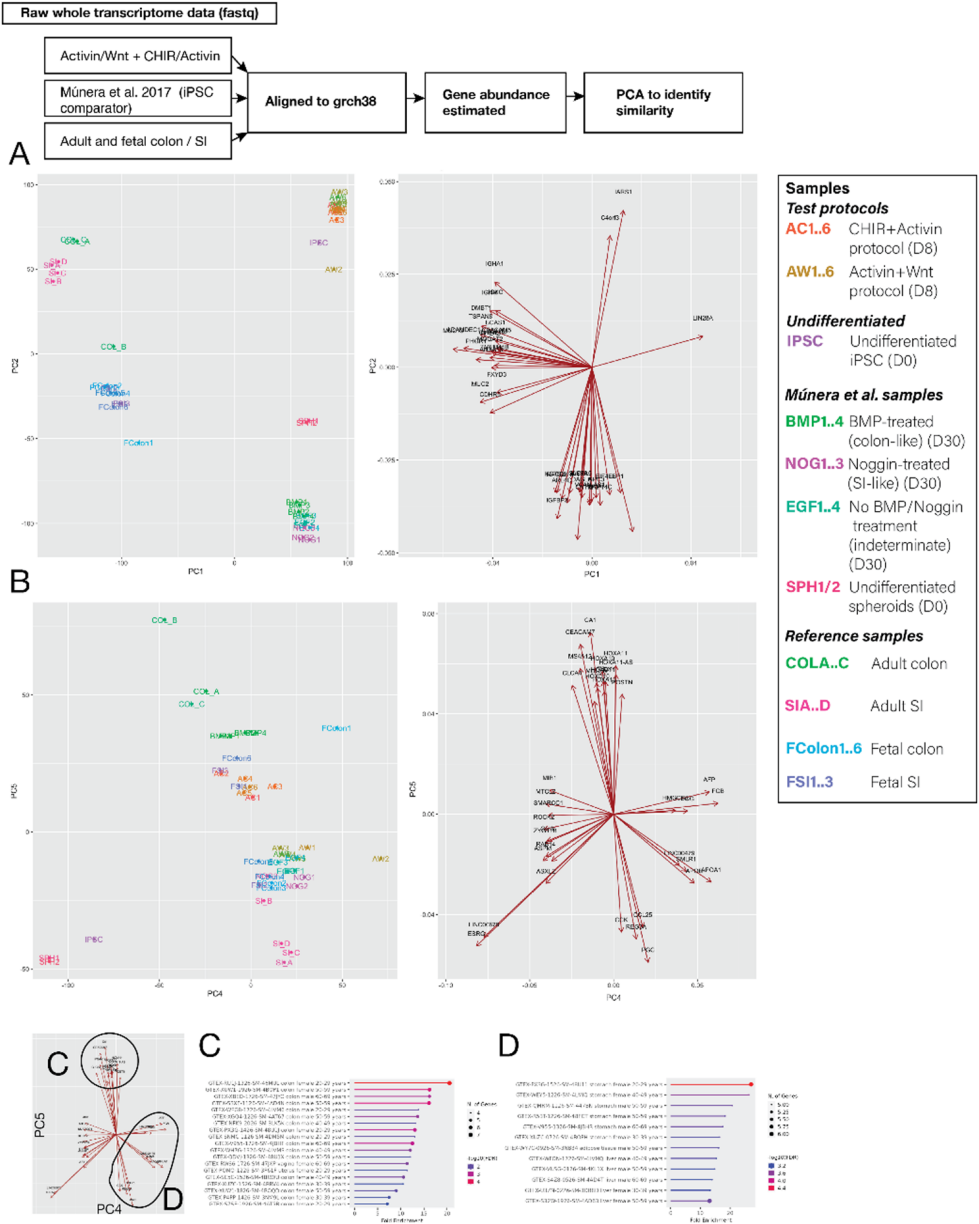
Whole transcriptomic comparison with alternative protocol and human tissue demonstrated colonic polarisation of CHACT-differentiated cells. Fastqc files were analysed from RNA sequencing experiments performed on undifferentiated iPSCs (*n* = 1), CHACT (AC; *n* = 6) and Act/Wnt (AW; *n* = 6) protocols at day 8, the samples described in Múnera et al. 2017 after hindgut induction protocol at day 7 (SPH, *n* = 2) and after 28 days in Matrigel with media either EGF only (EGF; *n* = 4), EGF + Noggin (NOG; *n* = 3) or EGF + BMP (BMP; *n* = 4) using datasets uploaded to ArrayExpress (see data availability for accession numbers). Data from tissue samples were from the same comparison used in Múnera et al. and consisted of adult colon (COL; *n* = 3), adult small intestine (SI; *n* = 4), fetal colon (FColon; *n* = 6) and fetal small intestine (FSI; *n* = 3) using datasets uploaded to ArrayExpress (see data availability for accession numbers). Raw fastqc files were trimmed and then gene counts estimated for each using Kallisto using human genome grch38. Kallisto abundance estimates were imported into R and then processed using principal component analysis to determine similarity between each sample. **A + B** Principal component analysis plots of PC1 and PC2 (**A**) and PC4 and PC5 (**B**). PC4 and PC5 showed a greater separation between small intestine and colon samples and were selected for further analysis. The legend shows the samples described above. **C + D** Gene set analysis of top gene loadings in PC4/PC5. Genes aligning with colon samples were first selected (**C**) and then gene overrepresentation analysis performed using ShinyGO with the GTEX.TissueExpressionUp gene set, derived from bulk tissue expression profiles of tissue. This showed that top genes aligning with colon in the PCA showed over-representation in colon samples. **D** Likewise, genes aligning with SI samples in the PCA showed over-representation of proximal gut tissues including liver, stomach and small intestine

We compared the bulk transcriptomic profiles after CHACT and Act-Wnt differentiation conditions with the transcriptomic profiles of cell populations obtained by Munera et al. using alternative serum-based protocols to generate colonic and small intestinal precursors, and with profiles obtained from fetal and adult tissues (Fig. 4). All hiPSC samples (CHACT and Act-Wnt protocols and those of Munera et al.) clustered closely on principal components 1 + 2 (Fig. 4A) likely reflecting immaturity in comparison with more established fetal and adult counterparts and the effects of different donors. Principal components 4 + 5 (Suppl. Figure 4B) showed that CHACT differentiated hiPSCs had profiles polarised towards colonic tissue samples similar to those treated with BMP in Munera et al. and resulted in colonic specification. Act-Wnt differentiated iPSCs had profiles overlapping with Noggin/EGF-only treated hiPSCs by Munera et al., suggesting polarisation towards the small intestine tissue samples. Gene set over-representation analysis of top-weight genes extracted from principal components 4 and 5 at colon (Fig. 4C) and small intestine (Fig. 4D) poles on the principal component analysis plot showed that these genes were associated with either distal gut (C) or proximal gut (D) tissue samples (GTEX.TissueExpressionUp gene set).

### From pluripotent stem cell to transplantable colon-like intestinal patches (CL-IPs) in fifteen days without residual pluripotency

**Fig. 5:**
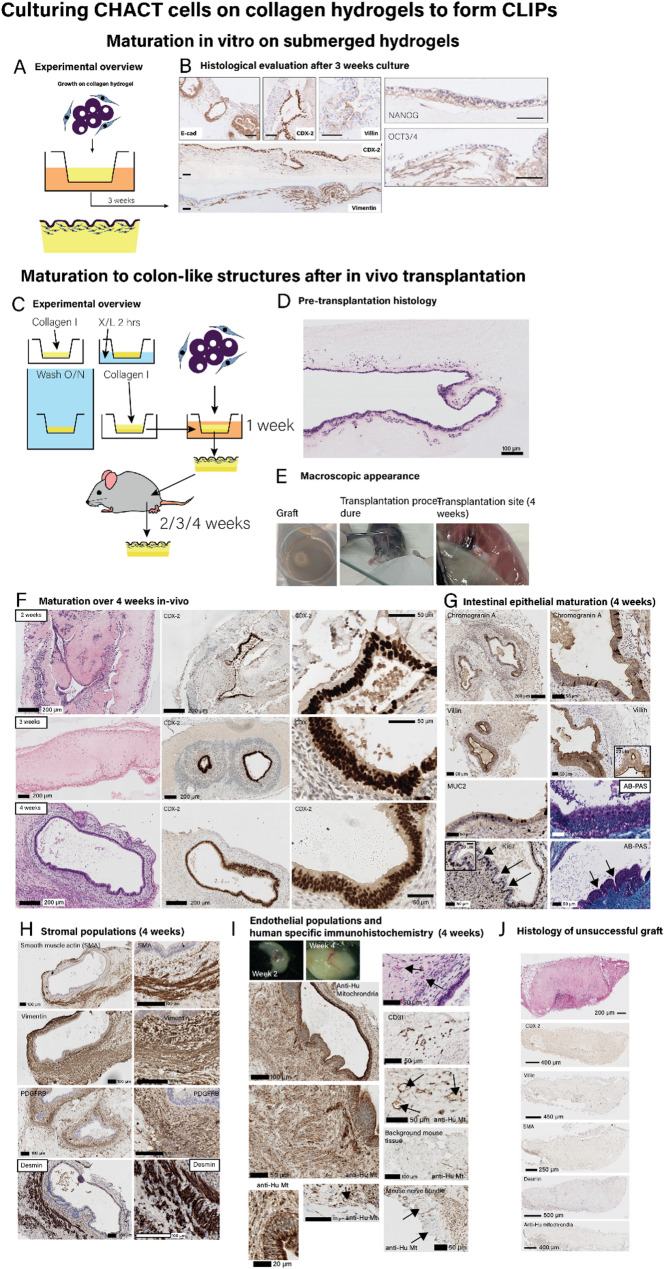
Culturing CHACT cells on collagen hydrogels to form colon-like intestinal patches (CLIPs). **A** Experimental overview of in vitro submerged collagen hydrogel culture (*n* = 10 males and *n* = 10 female mice). **B** Stratification into intestinal epithelium and mesenchymal self-assembled layers was shown when differentiated cells, as a mixed population, were seeded onto collagen hydrogels (2 mg mL^-1^) and cultured in vitro for 3 weeks and assessed for E-cadherin (E-cad), CDX-2, Villin-1, Vimentin, Nanog and Oct3/4 expression by immunohistochemistry, representative of *n* = 3 independent experiments. Scale bars represent 100 μm. **C** Experimental overview of in vivo experiments A base layer of collagen I hydrogel was formed in 12 well plate Transwells from 2 mg mL^-1^ neutralised collagen I and subsequently chemically cross-linked, after extensive washing, a further equal volume of collagen hydrogel (same concentration) was formed on top. Cells (mixed cell populations derived following the 8 day CHACT protocol) were then added and cultured for 1 week, before transplantation subcutaneously in immunosuppressed mice for 2–4 weeks. **D** Pre-transplantation histology of CoPs shows stratification into epithelium on the surface with underlying mesenchymal cells. **E** Macroscopic appearance of transplant before engraftment (top), during transplantation (middle) and at sacrifice (bottom). **F** Images showing graft maturation over 2–4 weeks showing H + E stained tissue (left column) and CDX-2 immunohistochemistry at low magnification (middle column) and high magnification (right column). **G** Intestinal epithelial maturation at 4 weeks demonstrated by immunohistochemistry for Chromogranin A, Villin, MUC2 and Ki67 and AB-PAS stain for mucus. Arrows indicate formation of colonic crypts. Insets show selected stains at high magnification. **H** Stromal cell maturation and organisation at 4 weeks assessed by immunohistochemistry for smooth muscle actin, vimentin, PDGFRB and desmin at low magnification (left column) and high magnification (right column). **I** Human origin of cell populations and functional human endothelium anastomosing with murine host shown by H + E stain, CD31 and human specific anti-human mitochondrial stain. Short arrows indicate vascular spaces of human origin, demonstrated by anti-human mitochondrial staining. Long arrows indicate mouse nerve bundle, with no staining by anti-human mitochondrial antibody. **J** Histology of grafts where no growth occurred after transplantation (unsuccessful grafts) was performed to demonstrate response to injury. In these cases, while the collagenous graft could be seen, no intestinal epithelial or mesenchymal tissue structures were seen, demonstrated by absence on H + E staining and undetectable expression of key lineage marker proteins by immunohistochemistry (CDX-2/Villin for intestinal epithelium, SMA/Desmin for intestinal mesenchyme and anti-human mitochondrial staining for human origin of cells)

From Day 9 onwards, co-differentiated cells (without MACS separation) were placed on collagen I hydrogels formed in Transwells to create “colon-like intestinal patches” (CL-IPs) (Fig. 5A) [28]. These colonic patches were intended to replicate the organisation of the colonic mucosa, with exposure of the culture surface to culture medium. By Day 22 of in vitro culture the epithelial cells in the CL-IPs lined the surface of the hydrogel and had assembled into crypt-like structures (Fig. 5B). These cells expressed the intestinal epithelial markers, E-cadherin and CDX-2, together with Villin-1 (which demonstrated notable polarised expression limited to the apical cell membrane). Vimentin-positive mesenchymal cells in CL-IPs were stratified beneath the surface epithelium and around the crypts. No evidence of residual pluripotent cells was demonstrated by immunohistochemistry for OCT3/4 and NANOG. Thus, our protocol facilitated the assembly of the co-differentiated population into colonic mucosa-like structures with a polarised epithelium supported by an underlying mesenchyme.

Given the wide array of cellular lineages seen among differentiated cells after Day 8 with the CHACT protocol, we wanted to determine whether tissue grafts formed from these cells – even at this early stage – were suitable for transplantation and were capable of further maturation towards recognisable intestinal tissue structures. Given transcriptomic findings suggestive of colonic differentiation, we were also interested to ascertain whether maturation polarised towards colonic vs. small intestinal tissue structures. Taking cognizance of the physical stresses of tissue grafting, the colon-like intestinal patches (CL-IPs) were modified by chemically cross-linking the lower part of a collagen gel (to increase strength for handling) while the upper part was not cross-linked (Fig. 5C); the mixed cell population was then seeded on top of the uncross-linked surface of the collagen ‘patch’ and cultured in vitro for a further seven days. After a further seven days of in vitro, submerged, static culture, cells were seen to have migrated into the collagen gel with a self-organised stratified epithelial surface Fig. 5D/E). These CL-IPs were transplanted into the sub-cutis of immunocompromised (*Rag2*^*−/−*^ *IL2RG*^*−/−*^) mice and harvested after 2, 3 or 4 weeks (Fig. 5F). Across all experiments we found viable tissues consistently in around 55% mice (*n* = 11 out of 20 mice) indicating high transplant efficiency, and comparing favourably with other approaches described in the literature [13].

In our transplants, there was progressive increase in tissue organisation and cell maturation and, by four weeks, luminal spaces lined by epithelium with concentric layers of specialised mesenchyme emerged (Fig. 5F). The epithelium showed intestinal differentiation including multiple cell types (neuroendocrine, goblet cells, and proliferative cells) (Fig. 5G). Underlying mesenchyme resembled lamina propria and smooth muscle fibres (identified by Smooth Muscle Actin (SMA) and desmin expression) formed a boundary beneath this, recapitulating *muscularis mucosae* (Fig. 5H). The tissues became highly vascularised and, using antibodies specific for human mitochondria, the vascular channels were shown to be lined with human-origin CD31 + endothelial cells (Fig. 5I). The vascular channels contained blood indicating that human blood vessels had undergone anastomosis with murine dermal blood vessels. For ethical reasons we did not conduct sham incision control experiments, but in graft conditions where no post-transplantation growth was observed, we undertook histology and immunohistochemistry for key lineage markers (Fig. 5J). We saw no evidence of intestinal epithelial (CDX-2 / Villin) or mesenchymal (SMA / Desmin) cell populations and background mouse tissue showed no human mitochondrial expression, providing further confirmation that all tissue structures seen were of human iPSC origin.

One concern with transplantation of hiPSC-derived tissues is the possibility of residual pluripotency leading to teratoma development. To confirm earlier molecular assays (Figs. 1/S1) that the risk of teratoma had been removed through differentiation, we tested the cell populations in a teratoma assay performed at Day 8 of the CHACT protocol. This assay was performed in immunocompromised mice, which would permit any remaining pluripotent cells to form teratomas. When undifferentiated hiPSCs were implanted into the mouse testicular subcapsular space (Fig.S2A), they formed large teratomas with all three germ layers represented (*n* = 12). In contrast, when co-differentiated hiPSCs (Fig.S2B) were implanted, the transplants underwent degeneration with fibrosis and dystrophic calcification indicating apoptosis (*n* = 12). Occasional intestinal glands were seen. No tumours formed and no other tissue type that may have derived from other germ layers was seen. These experiments indicate that differentiation fate specification is complete and there is no residual teratoma-forming activity following differentiation using the CHACT protocol. This model is relevant to human transplantation conditions where immunosuppression may be required for successful engraftment of tissue.

### Digital spatial profiling of transplanted colonic patches reveals maturation and development of gradients of normal biomarker expression along the crypt axis

The colonic mucosa is dynamic; epithelial cells migrate from crypt base to crypt apex over 3–5 days [10], differentiating from multipotent stem cells into terminally differentiated cells as they migrate [29]. Tissue homeostasis is maintained by gradients of growth factors and cell surface receptors and these can be quantified using Digital Spatial RNA Profiling (DSP), allowing gene expression to be quantified in small regions of tissue.

**Fig. 6:**
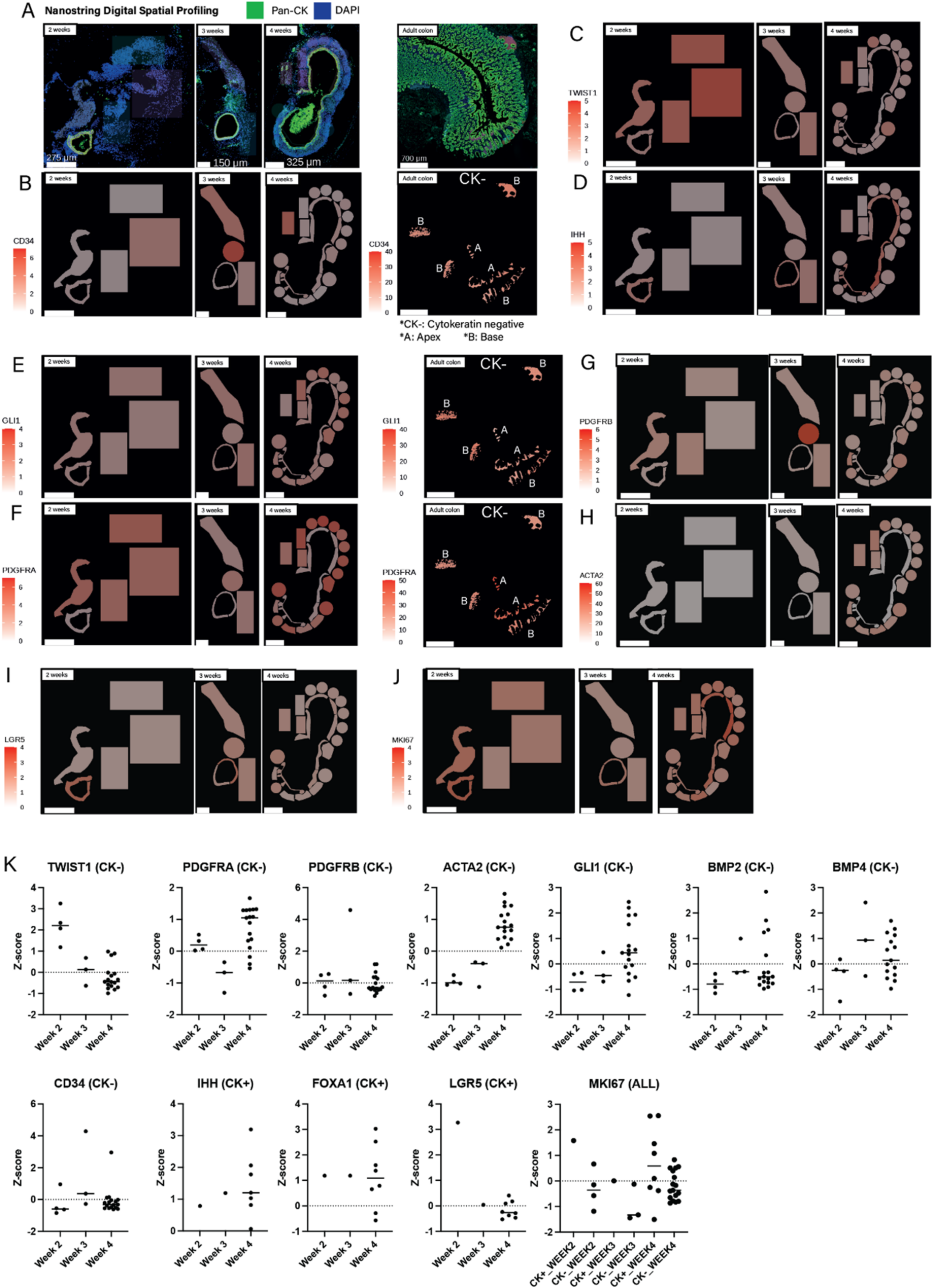
Nanostring Digital Spatial Profiling illustrating the maturation of cell populations within transplanted CoPs over several weeks of engraftment. **A** Pan-cytokeratin and DAPI stained iPSC-derived transplants at 2, 3 and 4 weeks and adult human colon control tissue (*n* = 1 for each timepoint). All markers below correspond to the same tissues. **B**–**J** Demonstration of endothelial transcripts and maturation of mesenchyme during engraftment. **B** Endothelial cell marker, *CD34*, expression correlates with presence of vascular spaces in in vivo grafts and normal adult colon(Adult colon – CK- = cytokeratin negative cells, A: apex of crypt, B: base of crypt). **C** Mesoderm marker, *TWIST1*, expression decreases across 2–4 weeks timepoints of in vivo growth demonstrating maturation of mesenchyme. **D** *IHH* expression is limited to the developing intestinal epithelium (**E**) *GLI1* (transducer of Hedgehog signalling) shows corresponding expression limited to the developing mesenchyme. GLI1 expression is higher in crypt base areas of illumination compared with crypt apices (**F**) *PDGFRA* shows increasing expression across 2–4 week timepoints of in vivo growth and is enriched crypt apex areas compared with crypt bases, suggesting mesenchymal *PDGFRA* expression may be required to drive more differentiated epithelial cell-types to emerge from in vivo hiPSC intestinal tissue grafts. Mesenchymal expression of (**G**) *PDGFRB* was constant over timepoints, while (**H**) *ACTA2* increased over time. Epithelial (**I**) *LGR5* decreased over time, consistent with increasing diversity of cell types seen at later timepoints. **J** *MKI67* expression was sustained across timepoints and compartments. **K** Z-scores were calculated from raw expression of each gene per AOI in each compartment and at each timepoint (CK+: cytokeratin positive [epithelial]; CK- cytokeratin negative [mesenchymal])

Transplanted CL-IPs morphologically resembled colonic mucosa, thus we sought to show that this was the case transcriptionally by comparing DSP data from histologically normal human colon with CL-IPs sampled at 2 weeks, 3 weeks and 4 weeks after transplantation (Fig. 6A). Endothelial transcripts were demonstrated in transplanted tissues (Fig. 6B). Mesenchyme showed a progressive transcriptional shift over timepoints from an “immature” phenotype to a more mature phenotype (e.g., decreasing *TWIST1*, mesoderm marker, Fig. 6C/K). The mesenchyme retained *GLI1* expression, important for maintaining the stem cell niche, while epithelium became enriched for Hedgehog ligand, *IHH* (Fig. 6D/E/K). Of particular note, was increasing mesenchymal *PDGFRA* expression over timepoints (Fig. 6F/K) which coincided with evidence of increasing epithelial maturation. Mesenchymal gene *PDGFRB* levels were relatively constant (Fig. 6G/K) while *ACTA2* (Fig. 6H/K) increased over time. BMP ligands *BMP2* and *BMP4* showed similar expression by mesenchyme over all timepoints. Epithelial *LGR5* decreased over time, consistent with increasing epithelial diversity seen on histology (Fig. 6I/K), while colonic lineage marker *FOXA1* was relatively constant (Fig. 6K). *MKI67* expression was constant across time and cell compartments demonstrating sustained proliferation and growth of transplanted cell populations (Fig. 6J/K). In normal adult colon tissue, we showed an opposing apex-base gradient of both *GLI1* (Fig. 6E) and *PDGFRA* (Fig. 6F) corroborating other studies which have demonstrated PDGFRA+ mesenchymal cells at the crypt apex [30]. Our data suggest that the emergence of this population may be important for intestinal epithelial maturation, as demonstrated recently by Huycke et al. [31]

## Discussion

Here we have developed a rapid colonic co-differentiation platform for human induced pluripotent stem cells. We show that these mature into complex multi-layered colonic structures when transplanted in vivo subcutaneously. Our platform overcomes some of the challenges associated with current protocols by using minimal media supplements and not requiring 3D organoid culture techniques before transplantation. The availability of hiPSCs raises the possibility of engineering therapeutic autologous cell transplants to replace damaged tissue, or to derive cell populations for use in in vitro tissue models accurately replicating the genetic background of patients with specific disease states. hiPSCs undergo differentiation into mature tissue derived from any or all the embryonic germ layers. To use hiPSCs as a cell source for the generation of healthy tissue, differentiation must be precisely controlled so that only the target tissue is produced. Furthermore, since a tissue rarely consists of only one cell type, all cell populations should be present and in the correct spatial location if the tissue is to be functional.

Unlike many published protocols, CHACT is serum- and antibiotic-free, facilitating potential translation into a clinically useful therapeutic. It has been replicated in several hiPSC lines and in different laboratories illustrating the robustness of the protocol. The CHACT protocol generates multiple intestinal cell lineages simultaneously; confirmed by single-cell mRNA sequencing as well as in vivo engraftment studies. Co-differentiated cell populations do not contain residual pluripotency and they have been grown on biocompatible collagen gels (already in routine use in surgical applications) to create CL-IPs.

Finding cell sources for replacement therapies has largely focussed on derivation from native tissues or stem cell sources from adult tissue. Intestinal stem cells isolated from adult tissue can be cultured in vitro as 3D organoids that mimic the function and structure of the original tissue. These 3D organoids are usually cultured in Matrigel^®^ or similar containing signalling factors important for the maintenance and regulation of the cells within the organoid [32]. Organoid cultures of intestinal stem cells have played an integral role in our understanding of intestinal stem cell biology and its regenerative potential [12] and the concept of organoid regenerative medicine has been explored previously in animal models of IBD. Related studies have demonstrated that replacement mucosal cells can integrate into mucosa and aid healing in the disease tissue [5] illustrating the potential of a cellular regenerative medicine therapy to support healing in IBD. To consider organoids as a cell therapy, organoids would have to be fully characterised requiring extensive optimisation, be able to undergo mass expansion in a controlled, time-efficient manner, and their efficacy in human disease would need to be demonstrated [3]. A further issue is that in diseased tissues it may not be possible to isolate healthy cells to then expand in cell culture. hiPSC-derived intestinal cell generation offers several advantages over a primary intestinal organoid approach, particularly that protocols can rapidly generate large numbers of cells and multiple cell lineages can be generated simultaneously. Other hiPSC studies have demonstrated some degree of co-differentiation but have considerably longer and more complex protocols including the use of serum, which we consider would limit clinical application [22]. Analysis of global transcriptomic profiles showed that by day 8, the CHACT protocol is capable of polarising cell populations towards colonic differentiation. Their colonic identity was reinforced by single-cell RNA profiling demonstrating that epithelial populations mapped to “distal progenitor” and “transit amplifying” at the day 8 stage, while mesenchymal populations showed a posterior HOX code. Compared with other published protocols, our method achieves rapid differentiation, with a greater diversity of cell types which form mature intestine. Many protocols require a further period of 3D culture encapsulated in extracellular matrix, creating further cost, complexity and limiting cell yield. Our method is simpler than others published in the literature in the number of steps and number of required media supplements (Suppl. Table 1) [17, 18, 33, 34]. We replicated our findings across five different iPSC lines, including at an independent centre and using a commercial iPSC line. While we observed some variation in markers, such as MUC2, we attribute this to donor biological heterogeneity and progress along the intestinal epithelial differentiation trajectory. This is explained by the fact that iPSCs/endoderm must first commit to an intestinal progenitor phenotype, followed by further differentiation to the diverse cell types contributing to the mature intestinal epithelium. Nonetheless, in later transplanted tissues, these progenitor populations were capable of forming diverse cell types such as absorptive cells, goblet cells and neuroendocrine cells, while maintaining a polarised stem cell/transit amplifying niche. Furthermore, core markers of intestinal epithelial and mesenchymal lineage commitment such as EPCAM, CDX-2, SOX6 and ACTA2 were similarly expressed across all the lines, and with a similar proportion of epithelial/mesenchymal cells in each. Thus, we provide robust support for our core finding that our protocol is capable of driving reproducible intestinal and mesenchymal lineage commitment. Our CHACT protocol makes use of defined media supplements, which in future could be substituted with GMP compliant xeno-free equivalents. While we intentionally excluded serum, as our requirements for this translational protocol altogether, we did benchmark the protocol against a widely used protocol in the literature as well as reference datasets [17] through transcriptomic comparison and showed our protocol produces similar results and demonstrates that early exposure to CHIR-99021 is capable of both polarising cells towards colonic identity as well as patterning multiple cell lineages. Our protocol does currently use animal-derived extracellular matrix as a substrate for growing cells and in future work we would anticipate optimising a fully-defined xeno-free alternative.

Our primary aim was to generate proof-of-concept tissue graft (CL-IPs), which could be easily transplantable. While we showed that our protocol was capable of generating organoids, we reasoned that a tissue graft with cells exposed to the surface would allow a greater number of cells to be transplanted and would be more clinically relevant to the intestine, where epithelial cells are exposed to luminal fluid. Thus, we focused on developing transplantable CL-IPs. To demonstrate maturation potential of cells, xenograft transplantation into immunosuppressed mice is regarded as a gold-standard method for both intestinal organoid and hiPSC-derived intestinal cells [13, 19]. Our subcutaneous model provides comparative data with other hiPSC-derived intestinal cells and primary intestinal organoid methods in which xenografts have been made at extra-intestinal sites including subcutis and renal capsule. While in this study, we did not transplant xenografts into the intestine, the ability of our hiPSC-derived tissues to mature into colonic tissues outside the intestine is additional testament to their committed colonic differentiation without tissue-specific microenvironment cues. Transplanted CL-IPs into the *subcutis* of mice showed development of colonic mucosa complete with human-derived *muscularis mucosae* and human-derived blood vessels that successfully anastomosed to murine blood vessels. The mucosa developed crypt structures and precursor epithelial buckling [35, 36] as well as diverse epithelial and mesenchymal populations. Markers such as Villin, MUC2 / AB-PAS demonstrated expected localisation towards the luminal surface and Ki-67 towards the base, in keeping with normal colonic crypt organisation. While grafts do not fully recapitulate all adult colonic structures, crypt organisation was more apparent in week 4 transplants compared with those at week 2, which were by contrast more primitive, demonstrating evidence of maturation. Notably, no villus structures were seen, which have been observed in previous studies deriving intestinal cell populations from hiPSCs [17] when cells were transplanted in vivo, supporting our transcriptomic evidence of colonic polarisation. Compared with previous hiPSC approaches [19, 37], we saw similar levels of epithelial, mesenchymal and endothelial maturation including ability to form smooth muscle bundles and anastomose with host vasculature, but with a shorter timeframe to transplantation from starting intestinal differentiation to transplantation (15 days vs. 37 days). Significantly, we showed that mesenchymal populations arose from hiPSC-derived CL-IPs (rather than host-cell infiltration) using highly specific anti-human mitochondrial antibody as well as human-specific probes on spatial transcriptomics. In future work we would consider it would be worthwhile to complement spatial transcriptomic studies presented here with single-cell RNAseq analysis on in vivo CL-IPs to investigate the full diversity of cell populations including rare cell populations.

We were able to be grow and mature our multi-lineage cells on the surface of hydrogel scaffolds to form CL-IPs, which we consider is likely to be more amenable to colonic transplantation in future. This scaffold model is similar to that developed by Meran et al. [13], where the authors were able to successfully transplant in vitro expanded primary intestine-derived cells on decellularized human jejunal tissue scaffolds (rather than colon) into the subcutis of mice. While we have not demonstrated direct colonic engraftment such as that shown by Yui et al. for organoids [38], we consider this a logical next step to test the behaviour of these cell populations in the intestinal microenvironment and their ability to heal diseased mucosa. Our approach of transplanting iPSC-derived tissue with established architecture is, however, different from that of Yui et al. and would require a specialised delivery mechanism, given that an enema of single-cells would likely lose any morphological cues. A similar contrast can be drawn with iPSC-based approaches that transplant whole organoids compared with tissue patches [17, 22]. We therefore consider our approach of generating pre-formed tissue with an open luminal surface to be at the state-of-the-art for iPSC-based intestine tissue engineering. We consider several advantages of an hiPSC approach in comparison as (i) hiPSC-derived cells could be grown on a relatively simple hydrogel platform and (ii) give rise to multiple populations of cells in a single protocol, compared with harvesting multiple cell populations. Our hiPSC-derived stromal populations replicate the full complexity of the intestinal stroma, as demonstrated by scRNA-seq studies [6, 7]. Thus, it follows that this may mediate appropriate epithelial crypt organisation, through Wnt and BMP signals, as well as releasing pro-angiogenic signals to promote vascularisation. This is exemplified by increased expression of *PDGFRA* over several weeks of transplantation, correlated to gradual acquisition of PDGFRA+ cells, which are known to drive intestinal epithelial differentiation [31]. Finally, we anticipate the use of hiPSCs using our CHACT differentiation protocol offers greater opportunity for scale-up manufacture of these cell populations, unlocking the cell supply chain to produce tissue grafts for engraftment to the bowel to support healing. Our goal in this study was to develop an early-stage and rapid platform to generate colonic tissue patches with potential for further development as a human therapeutic in future. While our differentiation strategy builds on established developmental paradigms, its advance lies in the reconfiguration of early signalling dynamics to enable coordinated epithelial, mesenchymal, and endothelial differentiation within a fully defined, serum-free system. By altering the timing of Wnt pathway activation and eliminating serum, we establish a scalable and clinically compatible protocol that reproducibly generates colonic-patterned tissue without reliance on undefined components. We consider that the technology will require further development to progress as a potential human therapeutic, to enable scaling-up to meet clinical needs for cell supply as well as stringent requirements for GMP-compliance. These findings support the notion that precise temporal control of signalling, rather than incremental factor optimization, is sufficient to drive multi-lineage intestinal specification. While understanding the fundamental developmental processes at play in early cell type specification, in particular the role of Wnt signalling in specifying endoderm and lateral plate mesoderm is important, we considered this beyond the scope of this study. Likewise, while individual growth factor contributions were not systematically investigated, the protocol was intentionally designed to prioritise robustness, scalability, and clinical compatibility over incremental optimization, consistent with its intended therapeutic application. In future, the development of this technology towards the investigation of drug and nutrient absorption would be a valuable goal to support more accurate pharmacological and physiological assays, using advanced cell culture techniques such as microfluidic devices to mimic luminal and vascular flows.

In summary, we have developed a simple serum-free co-differentiation platform that derives multi-lineage colon-like intestinal cell populations from hiPSCs which can then be used to form transplantable colonic patches (CL-IPs) in 15 days. This platform therefore provides a practical foundation for scalable intestinal tissue engineering and future cell-based therapeutic applications. Our co-differentiation and hydrogel platform also has potential to be further developed to model intestinal diseases using heterogeneous cell populations in 3D cultures that allow investigation of complex spatial cell interactions and responses to exogenous treatments.

## Methods

### Cell culture and cell lines

hiPSCs were generated within the Nottingham Biodiscovery Institute (formerly the Centre for Biomolecular Sciences), University of Nottingham from fibroblasts harvested by punch biopsy of axillary dermis, using methods previously described [39] and were gifted to this project by Professor Chris Denning, University of Nottingham. The REBLPAT line (male) was used for most studies from passage 27 and used for experiments between passage numbers 30 and 36. BE31 and BE32 hiPSC lines (female) [40] were also used for validation. hiPSC lines were approved and derived under University of Nottingham ethics committee number 09/H0408/74. In addition, OSTI GI4380 hiPSCs (male) were used at the University of Oxford and were approved for usage as part of the Cancer Research UK CORGI2 trial by the South Central/Oxford Research Ethics Committee (17/SC/0079). An additional commercial cell line (SCTi-003a) was purchased from Stem Cell Technologies and used for validation experiments. In contrast to our to other lines which were reprogrammed from fibroblasts, this line was re-programmed from circulating T-cells. Pluripotency was demonstrated by expression of pluripotency markers and lack of differentiation markers by protein/mRNA quantification. Cells were used within 10 passages of prior characterisation.

hiPSCs were cultured in Essential 8™ medium (Life Technologies, Bleiswijk, Netherlands) to maintain pluripotency, at 37 °C, 5% CO2 in a humidified incubator. Medium was changed daily by aspirating spent medium, washing once with phosphate-buffered saline (PBS) and replacing with fresh Essential 8™ medium. Cells were cultured for three to four days to reach at least 80% confluence before passaging. In order to passage, cells were washed once in PBS, after which TrypLE Select (Life Technologies) was added for up to 5 min, until cleavage between cells could be seen upon microscopy, but before complete detachment from the culture surface. TrypLE Select was then quickly, but carefully aspirated, fresh Essential 8™ medium containing 10µM Y-27632 dihydrochloride (ROCK-inhibitor [ROCKi]) was added and the detached cells counted. Upon passage, approximately 30,000 hiPSCs cm^− 2^ were added to each culture vessel, which had been previously coated with Matrigel^®^ at 11.1 µg cm^− 2^ for at least 90 min incubated under standard cell culture conditions. For the first 24 h of culture, cells were grown in medium containing 10 µM ROCKi.

Unless otherwise specified, all cell culture was performed using aseptic technique under normoxic conditions at 37 °C with 5% CO2 in a humidified incubator. Cells were cultured in antibiotic-free conditions. Cells were observed daily under microscopy to assess viability and morphology. All cells used were tested routinely for Mycoplasma at least monthly through the Nottingham Biodiscovery Institute testing programme. All media constituents and supplements are detailed in Supplementary Table 2.

### Co-differentiation to posterior endoderm and mesoderm

hiPSCs were detached and passaged to an appropriate plate coated with Matrigel^®^ (6-, 12- or 24-well; Corning) at a rate of approximately 30,000 cells cm-2, as described in Experimental Models. Cells were maintained for the first 24 h in Essential 8™ medium with 10 µM ROCKi to attach and proliferate as pluripotent stem cells and incubated under standard cell culture conditions.

After 24 h, once cells were approximately 50–75% confluent, each well was washed once with warm RPMI-1640 (Life Technologies). Basal differentiation medium, consisting of RPMI-1640 with Glutamax (Life Technologies), 1x B27 supplement (Life Technologies) and 1x non-essential amino acids (NEAA, Life Technologies), was used for all conditions. The conditions tested experimentally for their ability to induce posterior endoderm are detailed in the main text and were formed of basal differentiation medium with the following growth factor supplements (also detailed in supplementary Table 2), unless otherwise specified dissolved in 0.1% bovine serum albumin (BSA): CHIR-99,021 (dissolved in DMSO; R + D systems, Minneapolis, USA), recombinant human Wnt-3a (Chinese Hamster Ovary cell (CHO) derived; R + D systems), recombinant human/mouse/rat Activin-A (dissolved in 4mM HCl; E.coli derived; Peprotech). The CHIR/Activin condition consisted of 24 h of CHIR-99021 (10 µM) followed by Activin-A (100 ng/mL) for 3 days, with daily media changes to induce endoderm and mesoderm co-differentiation. Activin/Wnt condition was Activin-A (100 ng/nL) and Wnt-3a (50 ng/mL) for 4 days while Activin condition was Activin-A (100 ng/mL) only for 4 days (daily media changes). To induce hindgut differentiation following initial endoderm/mesoderm specification, CHIR-99021 (5 µM) was used for all conditions for 4 days with daily media changes. Cells were grown under standard cell culture conditions. The culture medium was fully replaced daily including supplements. Briefly, the old medium was aspirated and culture surfaced washed once with warm RPMI-1640. Fresh medium was added to the same volume on each day. Each differentiation condition lasted up to 8 days after the first 24 h, and assayed at various time points (24 h, 96 h and 192 h) as detailed in the main text.

### Cell freezing and thawing

hiPSCs were prepared cryopreservation by detachment by TrypLE Select, as described for routine passaging. After detachment, hiPSCs were resuspended in 10 mL Knockout Serum Replacement medium (KOSR; Gibco) and a sample of the suspension counted in duplicate using an automated cell counter with Trypan blue stain. Cells were centrifuged at 300 xg for 5 min, after which the supernatant was discarded. Cells were then re-suspended in a volume of KOSR to achieve a concentration of 2 × 10^6^ cells mL^− 1^. Cryovials were prepared by adding 500 µL of 2x solution of freezing medium, consisting of 20% DMSO in KOSR. Next, 500 µL of the cell suspension was added to each cryovial such that each cryovial contained approximately 1 × 10^6^ cells. Cryovials were placed in a Mr Frosty controlled freezing container (Nalgene) and then transferred to a -80 °C freezer overnight. Cryovials were subsequently transferred to long-term liquid nitrogen storage within 7 days.

To thaw hiPSCs, a T25 flask was coated with Matrigel^®^ as described for routine passage. hiPSCs were brought to 37 °C by placing the cryovial in a water bath briefly until thawing was observed. The cell suspension was aspirated from the cryovial and transferred dropwise slowly into 10mL Essential 8™ medium. The cell suspension was then centrifuged (300 xg, 5 min) and the supernatant discarded. The cell pellet was resuspended in 5 mL Essential 8™ medium with ROCK inhibitor (as described for routine passage) and transferred to the Matrigel^®^-coated T25 flask. Medium was replenished the following day with fresh Essential 8™ medium (without ROCK inhibitor) and cells were cultured as described for routine maintenance.

### MACs cell separation

Cells were detached as described above and resuspended in dissociation buffer consisting of dPBS with 1% bovine serum albumin (BSA) and 2mM ethylenediaminetetraacetic acid (EDTA). MACs separation was performed according to manufacturer’s instructions, outlined as follows. Up to 10^7^ cells were pelleted by centrifugation (300 xg, 8 min) and resuspended in dissociation buffer (100µL) with anti-EpCAM microbeads (25 µL, Miltenyi Biotec) and anti-FcR (25 µL, Miltenyi Biotec). Cells were incubated at 4 °C for 30 min and gently agitated every 10 min. The solution was then topped up to 1 mL using dissociation buffer followed by centrifugation to pellet the cells. The MACs stand and column (Miltenyi Biotec) were set up within a microbiological safety cabinet by attaching a MiniMacs™ magnetic separator (Miltenyi Biotec) to the stand and then inserting an MS column into the separator. The column was then primed with 500 µL dissociation buffer. After centrifugation, the cell pellet was resuspended in 500 µL and then added to cell column. The non-retained/flow-through cells (EpCAM-) were collected in a centrifuge tube. The column within the separator was washed 4 times with dissociation buffer (500 µL per wash). The column was then removed from the magnetic separator, 1 mL of dissociation buffer was added, and the column plunger was inserted to force retained cells (EpCAM+) into a fresh centrifuge tube. Collected cells were then washed by centrifugation twice. After the first centrifugation, if cells were being used for downstream analysis, they were resuspended in dPBS, if being used for further cell culture, cells were suspended in appropriate culture medium.

### Neutralisation of collagen

Type 1 rat-tail collagen (Life Technologies) was used at a stock concentration of 3 mg mL^− 1^. To achieve a concentration of 2 mg mL^− 1^ for each 1 mL of collagen gel required, 667 µL of stock was added to a tube on ice. To this, 100 µL of 10X PBS were added. To neutralise the solution, 0.025 µL of sterile 1 M sodium hydroxide were added for each microlitre of collagen. The solution was topped up to 1 mL with medium (DMEM/F12), which if neutralised correctly displayed a slight pink colour of the phenol red pH indicator.

### Culture on collagen hydrogels

A method for demonstrating the growth of posterior endoderm subsequent to initial differentiation was performed on collagen hydrogels using previously published methods [28]. The procedure was performed in 12-well Transwell^®^ plates (Corning) with 0.4 μm pore size membranes. The plates were prepared by adding 500 µL of ice-cold neutralised collagen (see above) into each Transwell^®^ insert. The plates were then incubated for 1–4 h in standard culture conditions, to allow the collagen to form a semi-solid gel. Basal maturation medium consisted of DMEM / F12 (1:1) with L-glutamine (Life Technologies), 1x B27, 1x N2 (Life Technologies). This was initially supplemented with CHIR-99021 5 µM, FGF-4 (Peprotech) 100 ng mL^− 1^ and recombinant human Noggin 100 ng mL^− 1^ (Peprotech), as well as IGF-1 (100 ng mL^− 1^, Peprotech) and FGF-2 (50 ng mL^− 1^, Peprotech). For the initial period of culture, all media were supplemented with ROCKi 10µM.

In the bottom of each well with a Transwell^®^ insert 1.5mL of supplemented basal maturation medium was added. Cells were detached and re-suspended in supplemented basal maturation medium; 500,000 cells (12-well plate) were added in 500 µL of medium onto the surface of each Transwell^®^ insert containing gelled collagen.

Cell medium was changed in both chambers every 3–4 days for the first two weeks, but with only IGF-1 and FGF-2 supplemented. Following this, the medium was aspirated from the top chamber and only replaced in the basal chamber, to create an air-liquid interface. Cultures were continued for up to 4 weeks, with media replacement in the bottom chamber every 3–4 days.

Following culture, medium was aspirated from the wells and replaced with 10% (*w/v*) neutral buffered formalin (NBF; Sigma Aldrich) to fix the tissues. Gels were carefully removed from the Transwell^®^ insert and allowed to float in formalin and fixation took place overnight at room temperature, for at least 18 h. After fixation, gels were lifted from the culture plate, blotted on tissue paper to remove excess formalin, bisected and then placed in a plastic mould. Low melting point agarose (Sigma Aldrich) was warmed to allow melting and was then laid over the gel within the plastic mould. The mould was then placed on ice to solidify the agarose. After solidifying, the agarose was trimmed and the embedded gel placed in a tissue cassette. The agarose/gel composites were fixed in NBF for a further 24 h, before automated tissue processing (Leica) to dehydrate and take the tissues to paraffin wax. In brief, tissue cassettes were loaded onto the automated tissue processor and submerged into 50% (*v/v*) methanol in distilled water. Tissue cassettes were moved to sequentially higher concentrations of alcohol (50%, 75%, 95% and 100% [*v/v*]) for two hours each and then through three 100% xylene baths for two hours each. Finally, tissue cassettes were submerged in molten paraffin wax for two hours before being removed from the processor for embedding.

Following processing, the agarose/gel composites were embedded cut edge facing down in paraffin wax and mounted onto a cassette. Sections of cooled tissue blocks were cut using a microtome (Leica) at 3–4 μm, floated and collected onto poly-L-lysine coated slides. Slides were then either stored to be used later or warmed to 50–60 °C to melt the tissues onto the slide, before being used in histological assays described in subsequent sections.

### Stromal modulation experiments

Stromal cells were cultured on Matrigel^®^ coated 6-well plates using standard coating parameters. The baseline media consisted of DMEM/F12 (1:1) with IGF-1 (100 ng mL^− 1^), FGF-2 (50 ng mL^− 1^) and PDGFbb (2 ng mL^− 1^). For TGF-β experiments, media was supplemented with TGF-β1 (1ng mL^− 1^, Peprotech) or A83-01 (Sigma Aldrich, 500 nM). Cultures were maintained for up to 6 days. The same conditions were used for 3D collagen cell cultures described elsewhere but for longer periods. For purmorphamine experiments, baseline media was supplemented with purmorphamine (10 µM, Sigma Aldrich) and cultures were maintained for up to 6 days.

### RNA extraction and quantification

All RNA was extracted and purified from 6-well or 12-well plates, using a total mammalian RNA mini-prep kit (Sigma Aldrich) according to manufacturer’s instructions. Following media aspiration, wells were washed with PBS once. RNA lysis buffer containing 1% 2-mercaptoethanol was added to wells and incubated for 2 min at room temperature and the lysate was transferred to an Eppendorf tube, on ice. After addition of an equal volume of 70% ethanol, the mixture was transferred to an RNA binding column, and all steps for purification of RNA were performed according to the kit protocol, including an ancillary on-column DNA digestion for 15 min (Sigma Aldrich). Following purification, RNA was quantified using a Nanodrop™ 2000 spectrophotometer (Life Technologies).

Up to 2 µg RNA was reverse transcribed to cDNA using the Omniscript RT Kit (Qiagen, Manchester, UK) or High-Capacity Reverse Transcription Kit (ThermoFisher) according to manufacturer’s protocol. In brief, per 20 µL reaction, up to 12.5 µL of RNA template was added to 7.5 µL mastermix. The mastermix consisted of 1 µL reverse-transcriptase enzyme, 2 µL 10X RT buffer, 2 µL RT random primers, 0.8 µL 25X dNTP mix (100 mM), 1 µL RNase inhibitor. All reactions were topped up to 20 µL with nuclease-free water.

All procedures were performed in a decontaminated UV-light PCR hood using nuclease free reagents and plastic consumables. Quantitative real-time PCR was carried out on the resulting cDNA. Reactions were prepared using PowerUp™ SYBR™ Green Master Mix (Applied Biosystems, Waltham, MA, USA) using custom primer pairs (Supplementary Table 3; Eurofins, Ebersburg, Germany).

Primers were custom designed such as to span an exon-exon junctions and with a PCR product size of 70–150 bp. All PCR sample preparation was performed in a UV-sterilised hood. Equal quantities of cDNA were added in each reaction series and at least 2 replicates per cDNA sample were performed, as well as appropriate negative controls. PCR was performed on ViiA™ 7 Real-Time PCR machine using a fast-cycling protocol consisting of an initial hold stage of two minutes at 50 °C (for activation of UNG), followed by five minutes at 95 °C (to activate hot-start Taq polymerase) followed by 40 cycles of denaturation for 1 s at 95 °C followed by annealing at an optimised temperature between 56 °C and 65 °C for 30 s, a separate extension step was not required. Fluorescence was read during the annealing step of each cycle. After cycling stages, a melt curve stage was included to verify the specificity of PCR amplification.

For RNA extraction and quantification performed at the University of Oxford (related to supplementary Fig. 2), the following procedure was used. RNeasy microkit (Qiagen, 74004) was used for RNA extraction. Extracted RNAs were incubated with DNase1 (ThermoFisher, EN0521) at 37 °C for 30 min, followed by a 10 min incubation with EDTA at 65 °C. High-Capacity cDNA Reverse Transcription Kit (Applied Biosystems, 4368814) was used to generate complementary DNA from total RNA. Quantitative real-time-PCR (qRT-PCR) was performed on LightCycler96 (Roche) with human Gapdh used as an endogenous control. The IDs of Taqman Gene expression assays (Applied Biosystems) used in this study are EpCAM (Hs00901885_m1), Cdx-2 (Hs01078080_m1), Sox17 (Hs00751752_s1), Nanog (Hs02387400_g1), Klf5 (Hs00156145_m1), Wnt2b (Hs00921614_m1), BMP4 (Hs00370078_m1), GAPDH (HS99999905-m1).

CT values for individual primers were compared with reference to a housekeeping gene using Livak’s 2^−ΔCT^ method or 2^−ΔΔCT^ method if an appropriate reference condition was available for comparison.

### Flow cytometry (EpCAM)

Cells were washed once with PBS and detached with brief TrypLE treatment. Following aspiration of the TrypLE, cells were resuspended in warm RPMI-1640 and transferred to Eppendorf tubes. The tubes were centrifuged at 300 xg for 5 min and then washed once with PBS, followed by further centrifugation. Reactions were performed in Eppendorf tubes and each step was followed by centrifugation at 300 xg for 5 min unless otherwise specified. Primary Anti-EpCAM APC conjugated antibody with isotype control was used for all experiments (Miltenyi Biotec, Surrey, United Kingdom). Cells were blocked in 3% (*w/v*) BSA in PBS for 15 min, followed by washing and addition of primary antibody diluted in 3% (*w/v*) BSA in PBS, with incubation for 30 min at 4 °C. Cells were analysed using either an FC500 or MoFlo (Beckman Coulter, Indianapolis, IN, USA) flow cytometer, with all procedures kindly optimised by the staff of the School of Life Sciences Flow Cytometry Facility, University of Nottingham (Dr David Onion and Mrs Nicola Croxall).

Data were analysed using the Kaluza Analysis software package (Beckman Coulter). Gating parameters on forward/side scatter were used consistently across all samples, and non-singlet cells were excluded from fluorescent intensity analyses to avoid overestimation. Negative and isotype controls were performed, which determined the threshold fluorescent intensity values for positive staining.

### Bulk mRNA sequencing

Cells lysates were prepared directly from plates and RNA was extracted as described earlier. After quantification by Nanodrop, samples were frozen and stored at -80 °C. Samples were diluted in nuclease-free water to obtain 2 µg total RNA in 20 µL sample volume. All samples were sent on dry ice via overnight courier to Novogene Limited for sample QC and subsequent library preparation and sequencing. Results were returned in raw FastQC format, BAM alignment files and raw and normalised count matrices for bioinformatic analysis. Subsequent analysis was performed using open-source SeqMonk and R software. Briefly, normalised gene matrices were generated using SeqMonk and initial hierarchical clustering was performed using dual approach based on intensity difference (based on log-transform) and DESeq2 packages (raw counts) to compare gene expression differences across multiple datasets. For comparison between two conditions, R was used to run DESeq2 on raw gene count matrices followed by Volcano plots based on log10 adjusted p-value and log2 fold change for individual genes. All DESeq2 analyses were performed with default parameters including correction for multiple hypothesis testing using the Benjamini-Hochberg method.

### Single-cell mRNA sequencing

Cells were dissociated as described earlier, but with a prolonged dissociation time of 8 min to ensure a fully dissociated single cell population. Cells were resuspended in dPBS with 1% (*w/v*) BSA to prevent intercellular adhesion. Cells were counted within the cell culture facility, using an automated counter, and resuspended at 1 million cells mL^− 1^. A second count was performed within the DeepSeq facility as described below.

Single cell 3’ whole transcriptome sequencing libraries were prepared from dissociated cell suspensions using the Chromium Next GEM Single Cell 3’ Library and Gel Bead Kit v3.1, the Chromium Next GEM Chip G Single Cell Kit and the Dual Index Kit TT Set A (10X Genomics; PN-1000147, PN-1000127 and PN-1000215). Cell counts and viability estimates were obtained using the LUNA-II Automated Cell Counter (Logos Biosystems), Trypan Blue Stain, 0.4% *(w/v)* and Luna Cell Counting Slides (Logos Biosystems; T13001 and L12001). Live cell counts were used to calculate cell input, rather than total cell count, as visual inspection of cell field on the LUNA II and the gating histogram, showed that > 90% of cells were viable and that the cell counter appeared to be counting some extracellular debris as non-viable cells. The number of input cells targeted was 3,300 cells per sample, with the aim of generating sequencing libraries from ~ 2,000 single cells. All steps, including GEM Generation and Barcoding, Post GEM-RT Cleanup and cDNA Amplification and Library Construction were performed according to the Chromium Next GEM Single Cell 3’ Library and Gel Bead Kit v3.1 User Guide, Rev B (CG000315).

Variable steps of this protocol included using 12 cycles of cDNA amplification and 8–12 cycles of library amplification. Amplified cDNA was quantified using the Qubit Fluorometer and the Qubit dsDNA HS Assay Kit (ThermoFisher Scientific; Q32854) and fragment length profiles were assessed using the Agilent 4200 TapeStation and Agilent High Sensitivity D5000 ScreenTape Assay (Agilent; 5067–5592 and 5067–5593). Completed sequencing libraries were quantified using the Qubit Fluorometer and the Qubit dsDNA HS Assay Kit and fragment length distributions assessed using the Agilent 4200 TapeStation and the High Sensitivity D1000 ScreenTape Assay (Agilent; 5067–5584, 5067–5585).

Libraries were pooled in equimolar amounts and the final library pool was quantified using the KAPA Library Quantification Kit for Illumina Platforms (Roche; KK4824). Libraries were sequenced on the Illumina NextSeq 500 over two NextSeq 500 High Output v2.5 150 cycle kits (Illumina; 20024907) to generate > 25,000 raw reads per cell for each sample, using custom sequencing run parameters described in the 10X protocol. Following sequencing, raw outputs from the sequencer were converted to FastQC files, aligned to the reference genome (hg38) and outputted to raw and filtered count matrices using the CellRanger (v6.1.1) pipeline from 10X genomics.

Individual analysis was performed using jupyter-lab tools on Ubuntu Linux using the following packages: Scanpy (1.8.2), anndata (0.7.8), UMAP (0.5.1), NumPy (1.20.3), SciPy (1.7.3), Pandas (1.5.2), scikit (1.1.3), statsmodels (0.12.2), igraph (0.10.8), PyNNDescent (0.5.5), scvelo, cellrank, MatPlotLib, Scrublet and CellTypist (1.6.2). Briefly, samples were imported and merged. Predicted doublets were removed using Scrublet using a threshold of 0.25. Basic filtering for gene detection included presence in at least 5 cells and all cells with a minimum of 200 and maximum of 7000 genes. Genes with greater than 20% mitochondrial reads were filtered out. Cells were normalised and log-transformed and highly variable genes were then identified. Normalised expression levels next underwent principal component analysis (PCA), nearest-neighbour analysis (neighbours = 15 and PCs = 10, optimised using the PCA elbow plot) and uniform mapping and approximation projection (UMAP), followed by clustering using the leiden algorithm (resolution = 0.3).

Next, clusters were annotated using unbiased CellTypist automated labelling. The “Developing_Human_Organs.pkl” model was selected as it is based on cells representing similar differentiation stages. Annotations were made over leiden clusters using the majority-voting method. Feature plots, dot plots and matrix plots (heatmaps) were generated using scanpy functions. The epithelial leiden cluster was extracted from the main anndata object and assessed using the “Cells_Intestinal_Tract.pkl” model within CellTypist.

### In vivo assays

BVA/FRAME/RSPCA/UFAW Refining Procedures for the Administration of Substances Working Group report, the NCRI Guidelines on Experimental Neoplasia, and NC3Rs Guidance for in vivo techniques were followed, as were the ARRIVE reporting guidelines. A total of 5 female and 17 male CD-1 NuNu mice at 6–7 weeks old were purchased from Charles River, (Margate, UK), and 5 female and 17 male Rag2^−/−^ Il2rg^-/-^ immunodeficient mice at 6–7 weeks old were purchased from Envigo, (Hillcrest), UK. Both strains were tested in order to identify if the different immune statuses of the strains had any effects on the resulting tissues.

It was essential to use only males for the testicular teratoma study, while equal numbers of males and females were used for the subcutaneous engraftment study. Numbers used per condition group were between 3 and 4. This was because the object of the studies was tissue generation and growth for analysis, rather than achieving statistical significance. Randomisation and blinding was not performed. The mice were maintained in individually ventilated cages (Tecniplast UK, Northampton, UK) within a barriered unit, illuminated by fluorescent lights set to provide a 12 h light–dark cycle (on 07.00, off 19.00), as recommended in the guidelines from the Home Office Animals (Scientific Procedures) Act 1986 (UK). The room was air-conditioned by a system designed to maintain an air temperature range of 21 ± 2 °C and a humidity of 55% ± 10%. During the study, the mice were housed in social groups, three per cage, with irradiated bedding and autoclaved nesting materials and environmental enrichment (Datesand UK, Stockport, UK). A sterile, irradiated 5V5R rodent diet (IPS Ltd., London, UK) and irradiated water (SLS, UK) were offered ad libitum. The animals’ conditions were monitored throughout the study by an experienced animal technician. After a week’s acclimatisation, the mice were initiated with cells or scaffold as detailed. At the scientific end point of the studies, the mice were killed by cervical dislocation and testes or scaffolds were removed under sterile conditions.

Animals were anaesthetised using a Ketamine (Ketaset, Animalcare, York, UK)/Medetomadine (Sedastart, Animalcare, York, UK) cocktail (Ketamine: 75 mg kg^− 1^ / Medetomadine: 1 mg kg^− 1^) injected subcutaneously and reversed using Atipamezole (Sedastop, Animalcare, York, UK) at 1 mg kg^− 1^ *s.c.*.

### In vivo teratoma assay

Undifferentiated hiPSCs and cells differentiated according to the novel protocol and protocol Act-Wnt were dissociated and pelleted by centrifugation at 300 xg for 5 min. For initial experiments, either one million or two million cells were implanted to optimise conditions. Either CD1 nude or Rag2^−/−^ Il2rg^−/−^ immunosuppressed mouse models were used for optimisation. In final conditions, one million cells were implanted into Rag2^−/−^ Il2rg^−/−^ mice. Cell pellets were transported on ice to the Biosupport Unit (BSU), University of Nottingham as well as thawed Matrigel ^®^ on ice. Immediately prior to implantation, 50 µL of Matrigel was added to the cell pellet and cells were resuspended within the matrix.

This cell suspension was then injected into the testicular subcapsular space. Mice were monitored by BSU staff throughout the period for weight and signs of ill health. Seven weeks after implantation, the mice were terminated by cervical dislocation and a post-mortem examination of the testicular space and abdominal cavity was undertaken. Any tumour tissue or evidence of cell growth was excised. All tissues were photographed and then placed in formalin in preparation for further histological examination.

### In vivo subcutaneous engraftment of populated collagen scaffolds

Tissue scaffolds were prepared from collagen following an optimised protocol in which 250 µL collagen (2 mg mL^− 1^) from rat’s tail was neutralised to form a hydrogel within a Transwell ^®^ mould. After gelation, collagen gels were chemically crosslinked using a protocol adapted from Kim et al. [41]. Briefly, in a Transwell, 1.5 mL of N-hydroxysuccinimide (0.05 M), 1-ethyl-3-(3-dimethylaminopropyl)-carbodiimide (0.2 M) and 2-(N-morpholino)ethanesulphonic acid (0.033 M) equilibrated to pH 5 in water was added to the bottom chamber, with 0.5 mL of PBS in the top chamber, and incubated at room temperate for 1 h. Crosslinking was performed in order to strengthen the gel for handling during transplantation. After extensive washing, a further 250 µL of neutralised collagen (2 mg mL^− 1^) was added to the surface of the crosslinked collagen and allowed to gel. Next, 500,000 hiPSC-derived intestinal cells at day 8 were passaged to the surface of the gel in the presence of CHIR (5 µM), Noggin (100 ng mL^− 1^), ROCKi (10 µM), IGF-1 (100 ng mL^− 1^) and FGF2 (50 ng mL^− 1^) in DMEM/F12 medium supplemented with B27 and N2 supplements. The following day, medium was replaced with basal medium with additional IGF-1, FGF-2 and Rspo-1 (50 ng mL^− 1^). Medium was replaced again on day 4 and then on day 6 (one day before implantation). This culture medium contained IGF1 (200 ng mL^− 1^), FGF-2, Rspo-1 and Wnt-3a (50 ng mL^− 1^) to enhance to proliferation of intestinal epithelial stem cells.

On the day of transplantation, tissue grafts were carefully lifted out of the Transwell and placed in medium with the same constituents as well as ROCKi (10µM) to improve cell viability during engraftment. Tissues were engrafted by making a small incision into the flank of Rag2^−/−^ Il2rg^−/−^ immunosuppressed mice within a sterile hood. The tissue graft was inserted into the pouch formed by incision, and the incision was closed with a surgical clip. After two, three or four weeks, mice were terminated by cervical dislocation and the surgical site opened. Tissue from the site was removed and photographed, and fixed in formalin prior to histological examination.

### NanoString GeoMx DSP RNA profiling

Slides for NanoString analysis were prepared from formalin-fixed paraffin-embedded tissues. Multiple tissues were combined on a single slide to allow simultaneous analysis. NanoString data was collected from two separate slides consisting of representative tissues collected from different in vivo transplantation timepoints (slide1) and a reference normal adult human colon. Each NanoString slide consisted of two to six different pieces of tissue.

Slides were stained with pan-cytokeratin (NanoString) and DAPI stains and then hybridised with Cancer Transcriptome Atlas probes. The slides were kept hydrated at all times. The slides were then loaded onto the NanoString GeoMx data spatial profiling (DSP) instrument. Areas of illumination (AOI) were selected based on areas of relevant morphology, such as apical and basal crypt regions. Automated segmentation was also performed based on cytokeratin expression. Each AOI was selected to contain at least 100 predicted cells (nuclei) based on DAPI staining. Having selected the AOIs for each slide, automated harvesting was performed on the DSP machine and probes were collected in 96 well plates each with well-specific barcodes. Library preparation was performed according to manufacturer’s instructions and the pre-made libraries were sequenced by NovoGene Limited using Illumina NovoSeq 6000 using a PE-150 sequencing strategy on a single flow-cell lane. After sequencing, fastq files were loaded into the DSP instrument for pairing with AOIs.

To process the NanoString data, we utilised the SpatialDecon package. Prior to deconvolution, we generated cell type expression profiles from the integrated foetal gut cell atlas object, considering cells with more than 10 genes and cell types with more than 5 cells. Background counts were determined using negative probes, and the SpatialDecon algorithm was applied to estimate cell type abundances for each AOI.

Subsequently, we employed the SpatialOmicsOverlay package to visualise the data on the image data. Initially, we made necessary modifications to package functions to resolve compatibility issues. We then imported the image and annotation data along with counts into the SpatialOverlay object. AOIs were grouped based on gene expression profiles using hierarchical clustering to identify similarities, resulting in four main clusters. Then, the cell types were assigned to each AOI based on the highest cell type abundance. Since each main slide consisted of 2 to 6 different sub slides, AOIs from each main slide were annotated according to their location, such as top-right or bottom-left. To focus on individual sub slides, the SpatialOverlay object was cropped accordingly, and marker expression levels were visualised by plotting them onto the AOIs.

### Basic histology

Slides were dewaxed and rehydrated by sequential immersion in xylene, methanol and distilled water. After rehydration, slides were laid flat and tissue sections were covered with Shandon instant haematoxylin (Life Technologies) for 3 min. Slides were then washed in distilled water, differentiated as necessary by rapid immersion and emersion in acid-alcohol (3% HCl in 95% ethanol) and washed again in distilled water. Submersion in Scott’s tap water (1% w/v magnesium sulphate and 0.067% w/v sodium bicarbonate in distilled water) was performed for 5 min to blue the haematoxylin, followed by further washing in distilled water. The slides were stained with Shandon instant eosin (Life Technologies) for 30 s, briefly washed in distilled water and then dehydrated by sequential immersion in ethanol and xylene. Slides were mounted with DPX and a coverslip was placed over the tissue.

For Alcian blue staining, tissues were similarly rehydrated. Slides were incubated in 3% (*v/v*) acetic acid solution for 1 min to acidify the tissue and then immersed in Alcian blue solution for 15 min. After washing in distilled water, slides were differentiated as necessary in acid-alcohol, washed in distilled water and then tissues were covered with nuclear fast red solution (Sigma Aldrich) for 5 min for counterstaining. Following washing, slides were dehydrated and mounted as described above.

### Immunohistochemistry

Slides were dewaxed and rehydrated as described above. Owing to previous fixation, antigen retrieval was performed by incubating slides in simmering sodium citrate buffer (pH6) for 20 min; a microwave set on low power was used to maintain temperature (approximately 95 °C). Once cooled, slides were mounted under water onto Shandon Sequenza^®^ coverplates (Life Technologies) and placed into a Sequenza^®^ rack. Slides were washed three times with 200 µL TBST (Tris-buffered saline with 0.01% Tween-20).

All immunohistochemistry was performed using the Novolink™ polymer detection system according to manufacturer’s instructions; all volumes used were 100 µL and washing was performed with TBST. Antibodies and conditions are detailed in Supplementary Table 4. Briefly, following protein and peroxidase blocking steps and washing, 100 µL of diluted antibody solution (in TBST) was added per slide; see Suppl. Table 4 for details of antibodies used and concentrations. Following overnight incubation in the primary antibody solution at 4°C, slides were washed and exposed to the post-primary solution for 1 hour at RT, polymer-peroxidase for 30 minutes, 3’,3’-diaminobenzidine (DAB) for 5 min and modified haematoxylin for 5 min. After this, slides were dehydrated and mounted as described above. Some IHC was performed by the Nottingham University Hospitals Cellular Pathology Department, according to standard operating procedures, which are available upon request; any such assays are indicated within the text.

### mRNA in situ hybridization

To detect expression of stem cell marker *LGR5* in the cultured cells on day-08 of the differentiation, EpCAM^+^ cells were fixed in 4% paraformaldehyde for 30 min at RT on a glass slides and dehydrated through ascending concentrations of ethanol (50%, 70%, 100%) and stored at -20 C. On the day of the staining, the cells were rehydrated by submerging the slides in 70% ethanol (2 min), 50% ethanol (2 min) and 1X PBS (10 min) at RT. The cells were then perforated by rinsing the slides in 1X PBST (1X PBS containing 0.1% Tween20) for 10 min at RT. After washing in 1X PBS (5 min), cells were incubated for 10 min at RT in RNAscope hydrogen peroxide solution (Cat # 322335). Then, slides were washed in 1X PBS and incubated in RNAscope Protease III solution, diluted 1:15 in 1X PBS, for 10 min at RT inside a humidity control chamber. After washing the slides in 1X PBS again, the manufacturer’s protocol for RNAscope Multiplex Fluorescent Kit v2 (Cat # 323110) was followed starting from the probe hybridisation step. *LGR5* mRNA signal was detected by using RNAscope catalogue probe 584,631.

To detect *LGR5* mRNA expression in formalin-fixed paraffin-embedded organoids, 4 μm thin sections were stained with RNAscope *LGR5* probe (Cat# 584631), following the manufacturer’s guideline, using RNAscope Multiplex Fluorescent Kit v2 (Cat # 323110). For the co-detection of *LGR5* mRNA, EPCAM and KI67 proteins, an immunofluorescence staining was performed after mRNA ISH following the protocols described by Montazid et al. 

(https://www.nature.com/articles/s41467-023-44138-6). Concentrations of antibodies used in this protocol were: 1:500 for EpCAM (Abcam, ab71916) and 1:500 for Ki67 (Cell Signaling, 12202).

### Statistical analysis

Statistical analyses were performed if appropriate to determine statistically significant differences. Normality of data distribution was tested using the Shapiro-Wilk test and if non-significant the distribution considered parametric. The student’s t-test (unpaired, two-tailed) or one-way ANOVA tests were used for continuous data. Unless otherwise specified, the following symbols are used to indicate statistical significance: * *p* < 0.05, ** *p* < 0.01, *** *p* < 0.001, **** *p* < 0.0001. Numbers of replicates, statistical tests and summary statistics are indicated in the methods and results sections or figure legends.

All statistical analyses and graphs were performed and produced using the following software packages: Microsoft Office 365 Excel, GraphPad Prism 9, R x86 v4.0.3/4.1.1/4.3 and Jupyter-lab v3.3.0. Image analysis was performed using Leica LASX, ImageJ/Fiji, QuPath and Adobe Photoshop.

## Supplementary Information

Below is the link to the electronic supplementary material.

Supplementary Material 1.

Supplementary Material 2.

## Data Availability

All raw and processed sequencing data are available on EMBL Array Express under the following accession numbers: bulk RNA-seq (related to Fig. 1 C/D): E-MTAB-13785 and E-MTAB-13799, single-cell RNA-seq (related to Fig. 3: E-MTAB-13845) and NanoString GeoMX DSP (related to Fig.5: E-MTAB-13834). Data for Suppl. Fig 2 was downloaded from EMBL Array Express: Múnera et al., 2017, accession number: E-MTAB-5658; adult SI/colon: accession number E-MTAB-1733, sample IDs: colon–ERR315348, ERR315357, ERR315484, SI–ERR315344, ERR315409, ERR315442, ERR315461. Fetal datasets were downloaded from the Gene Expression Omnibus (GEO) accession series GSE18927–the following sample IDs were used: fetal colon - SRR643745, SRR643748, SRR643755, SRR643757, SRR643759, SRR643761 and fetal SI – SRR643746, SRR643747 and SRR643760. Further information and requests for resources and reagents should be directed to and will be fulfilled by the lead contact, William Dalleywater (William.dalleywater@nottingham.ac.uk).

## Materials availability

This study did not generate any new or unique reagents.

## Code availability

All code is available at http://github.com/dalleywater/NUGut.
