# Supplementary material for: Generation of biologically responsive colon-like intestinal tissue patches from human induced pluripotent stem cells using a rapid co-differentiation platform
Source: Stem Cell Res Ther. 2026 Apr 9;17:182. doi: 10.1186/s13287-026-05006-4 (PMC13173943; doi:10.1186/s13287-026-05006-4)
Supplement: Supplementary file 1 — Supplementary Material 1. [file 13287_2026_5006_MOESM1_ESM.docx]

**Supplementary tables**

**Suppl. Table 1: Comparison of differentiation protocols**

| **Authors / Year** | | | | **Protocol summary** | **Cell types** |
| --- | --- | --- | --- | --- | --- |
| Qiu et al. 2021 | | | | Day 0: 1% DMSO  Day 1/2: Activin-A, CHIR-99021, PI-103  Day 3-7: Activin-A, FGF2 Penicillin/Streptomycin in media. | Endoderm/  Enterocytes/ Hepatocytes  Novelty: co-  differentiation of  enterocytes and hepatocytes |
| Macedo et al., 2018 | | | | Day 0 – 4: Activin-A (100ng mL-1)  Day 5 – 19: Bio (GSK-3β inhibitor) and DAPT  Day 20 – 35: Bio, DAPT, SB431542 (TGF-β  inhibitor), EGF and Wnt-3a Penicillin/Streptomycin in media. | Enterocytes Novelty:  differentiation of pure enterocyte populations, alternative to Caco-2  for barrier studies. |
| Múnera et al. 2017 | | | | Day 0 – 3: Activin A (100ng mL-1), RPMI-1640 with increasing concentrations of defined FBS (0 – 2%).  Day 3 – 7: FGF-4 (500ng mL-1) and CHIR-99021  (3µM), RPMI-1640 with 2% defined FBS and Activin-A (100ng mL^-1^) Penicillin/Streptomycin in media to induce spheroids.  Spheroids further cultured for 28 days with twice-weekly media changes in: DMEM/F12, N2, B27, 15mM HEPES, 2mM L-glutamien and EGF (100ng mL^-1^) only, EGF + Noggin (100ng mL^-1^) or EGF + BMP (100ng mL^-1^). | Intestinal endoderm and mesenchyme – either small intestinal or colonic.  Novelty: colonic patterning induced by BMP, while SI patterning induced by EGF / Noggin. |
| Tamminen et al. 2015 | | | | Day 0 – 4: Activin-A (100ng mL-1), Wnt-3a (75ng mL- 1), 1mM sodium butyrate, 2% B-27, RPMI-1640.  Day 5 – 8: Wnt-3a / FGF-4 / CHIR-99021 (varying), DMEM/F12 with 2% defined FBS. Penicillin/Streptomycin in media. | Intestinal endoderm / organoids  Novelty: reducing requirement of FGF-4  in media for intestinal differentiation. |
| Spence et al. 2011 | | | | Day 0 – 3: Activin A (100ng mL-1), RPMI-1640 with increasing concentrations of defined FBS (0 – 2%).  Day 3 – 7: Wnt-3a (50 or 500ng mL-1) and FGF-4 (50 or 500 ng mL-1) in DMEM/F12 with 2% defined FBS. Penicillin/Streptomycin in media. | Intestinal endoderm / organoids  Novelty: one of the first iPSC intestinal differentiation  protocols. |
| Watson et al. 2014 | | | | Day 0 – 3: Activin A (100ng mL-1), RPMI-1640 with increasing concentrations of defined FBS (0 – 2%).  Day 3 – 7: FGF-4 (500ng mL-1) and CHIR-99021  (3µM), DMEM/F12 with 2% defined FBS. Penicillin/Streptomycin in media. | Intestinal endoderm / organoids, implanted in vivo  Novelty: extension of previous work, showed differentiation to mature structures with implantation into  renal subcapsule. |
| Sarvestani et al., 2021 | | | | Day 0 – 3: Activin A (100ng mL-1), RPMI-1640 with increasing concentrations of defined FBS (0 – 2%).  Day 3 – 7: FGF-4 (500ng mL-1) and CHIR-99021  (2µM), RPMI-1640 with 2% defined FBS. Penicillin/Streptomycin in media. | Intestinal endoderm and mesenchyme, cultured as organoids and implanted in vivo Novelty: extension of previous work in Spence lab, showed changes in  mesenchyme composition in iPSC  from IBD patients. |
| Kitano et al. 2017 | | | | Day 0 – 3: Activin A (100ng mL-1), RPMI-1640 with increasing concentrations of defined FBS (0 – 2%).  Day 3 – 7: FGF-4 (500ng mL-1) and CHIR-99021  (3µM), RPMI-1640 with 2% defined FBS. Primocin in media. | Intestinal endoderm / organoids, seeded onto decellularized intestine and implanted in vivo.  Novelty: protocol  similar to that published by Spence et al., but application  to decellularized intestine. |
| Takahashi | | et al. | 2018 | Day 0 – 1: Activin A (100ng mL-1), Wnt-3a (20ng ml- 1), RPMI-1640.  Day 1 – 2: Activin A (100ng mL-1), FGF-2 (8ng mL-1), RPMI-1640 with 0.2% defined FBS.  Day 2 – 3: Activin A (100ng mL-1), FGF-2 (8ng mL-1), RPMI-1640 with 2% defined FBS.  Day 3 – 7: FGF-4 (500 ng mL-1), Wnt-3a (500ng mL-  1), RPMI-1640 with 2% defined FBS. Penicillin/Streptomycin in media. | Intestinal endoderm / organoids.  Novelty: found that Wnt-3a/FGF-2 levels during endoderm formation can improve efficiency of formation. |
| Ogaki | et | al. | 2015 | Day 0 – 1: 10% FBS, NEAA, 2-mercaptoethanol (100 | Intestinal |
|  |  |  |  | µM), DMEM. | differentiation to |
|  |  |  |  | Day 1 – 5: Activin A (0 – 100ng mL-1), 2% B-27, 0 – | organoids. Endoderm |
|  |  |  |  | 1.6% DMSO, NEAA and 2-mercaptoethanol (100 | also differentiated to |
|  |  |  |  | µM), DMEM. | hepatic and pancreatic |
|  |  |  |  | Day 5 – 11: BIO (5µM), DAPT (10 µM), 10% KSR, | lineages with |
|  |  |  |  | NEAA, 2-mercaptoethanol (100 µM). | alternative protocol |
|  |  |  |  | Penicillin/Streptomycin in media. | from day 5 onwards. |
|  |  |  |  |  | Novelty: use of |
|  |  |  |  |  | DMSO as adjunct to |
|  |  |  |  |  | differentiation/reduce |
|  |  |  |  |  | requirement for |
|  |  |  |  |  | Activin A. |
| Mithal | et | al. | 2020 | Day 0 – 2: StemDiff definitive endoderm kit (Stem Cell | Mesenchyme-free |
|  |  |  |  | Technologies) | organoids. |
|  |  |  |  | Day 2 – 3: Dorsomorphin (2 µM) and SB431542 (10 | Novelty: highly |
|  |  |  |  | µM) with Y27632 (ROCK inhibitor) in base medium | efficient derivation of |
|  |  |  |  | [DMEM/F12 3:1, 2% B-27, 1% N2, 0.1% ascorbic | lung and intestinal |
|  |  |  |  | acid, 1% glutamax, 2% BSA, 0.1% Primocin] | organoids with little |
|  |  |  |  | Day 3 – 5: As for day 2 – 3, without Y27632 | non-endoderm |
|  |  |  |  | Day 5 – 15: CHIR-99021 (3µM), BMP-4 (10ng/mL), | differentiation, note: |
|  |  |  |  | retinoic acid (100nM) in base medium. | use of commercial kit |
|  |  |  |  | Note: Primocin in media. | for endoderm |
|  |  |  |  |  | induction and so |
|  |  |  |  |  | constituents |
|  |  |  |  |  | unknown. |
| Saari et al. 2022 | | | | Day 0 – 1: Activin A (100ng mL-1) and CHIR-99021 | Multi-lineage |
|  | | | | (3µM), RPMI-1640. | endoderm |
|  | | | | Day 1 – 2: Activin A (100ng mL-1), 0.8% KSR, RPMI- | differentiation |
|  | | | | 1640. | potential (intestine, |
|  | | | | Day 2 – 3: Activin A (100ng mL-1), 8% KSR, RPMI- | pancreas, hepatic and |
|  | | | | 1640. | lung). Novelty: Large |
|  | | | | Day 3 – 7: replated and cultured in CHIR-99021 (3 | scale production of |
|  | | | | µM), FGF-4 (500ng mL-1), 2% FBS (experimental), | cells in stirred |
|  | | | | RPMI-1640. | bioreactors. |
|  | | | | Penicillin/Streptomycin in media. | Endoderm |
|  | | | |  | differentiation is |
|  | | | |  | xeno-free / |
|  | | | |  | chemically defined, |
|  | | | |  | though intestinal |
|  | | | |  | protocol uses FBS. |

**Suppl. Table 2:**

**Culture medium and supplements**

| **Growth factor name** | **Manufacturer** | **Standard Concentration** | **Source** |
| --- | --- | --- | --- |
| IGF-1 | Peprotech | 100ng/mL | E.coli |
| FGF-2 | Peprotech | 50ng/mL | E.coli |
| FGF-4 | Peprotech | 500ng/mL | E.coli |
| Noggin | Peprotech | 100ng/mL | E.coli |
| Rspondin-1 | Peprotech | 50ng/mL | CHO* |
| Activin-A | Peprotech | 100ng/mL | CHO |
| PDGF-BB | Peprotech | 2ng/mL | E.coli |
| TGF-β1 | Peprotech | 1ng/mL | HEK293* |
| Wnt-3a | R&D systems | 50ng/mL | CHO |
| EGF | R&D systems | n/a | E.coli |
| CHIR-99021 | Biotechne | 5-10µM | Synthetic |
| A83-01 | Sigma Aldrich | 500nM | Synthetic |
| Purmorphamine | Sigma Aldrich | 10µM | Synthetic |
| ROCK inhibitor (Y-27632 dihydrochloride) | Tocris | 10µM | Synthetic |
| B27 supplement | Life Technologies | 1X | Complex |
| N2 supplement | Life Technologies | 1X | Complex |
| Non-essential amino acids | Life Technologies | 1X | Complex |
| RPMI-1640 + Glutamax | Life Technologies | N/A | N/A |
| DMEM/F12 1:1 + HEPES | Life Technologies | N/A | N/A |
| Essential 8™ medium | Life Technologies | N/A | Synthetic |

**Suppl. Table 3: qRT-PCR primers**

| Primer name | Sequence 5’ – 3’ |
| --- | --- |
| CDX2 | Fwd: GGCAGCCAAGTGAAAACCA  Rvs: AGCGACTGTAGTGAAACTCCT |
| NANOG | Fwd: CAATGGTGTGACGCAGGGAT  Rvs: TGCACCAGGTCTGAGTGTTC |
| POU5F1 | Fwd: CAAAGCAGAAACCCTCGTGC  Rvs: CTCGGACCACATCCTTCTCG |
| SOX17 | Fwd: CGGGGACATGAAGGTGAAGG  Rvs: ACGACTTGCCCAGCATCTTG |
| FOXA2 | Fwd: GGGAGCGGTGAAGATGGA  Rvs: TCATGTTGCTCACGGAGGAGTA |
| T (Brachyury) | Fwd: GCTCACCAACAAGCTCAACG  Rvs: AGTTGTCAGAATAGGATTGGGAG |
| VIL1 (Villin) | Fwd: TGGTGTGGGAAGGGTTGTAG  Rvs: GGGGGTGATGACCAGGTTTT |
| SOX6 | Fwd: TAAGCAACTGATGAGGTCTC  Rvs: AGGCGATGGTGTGGTAGTT |
| WNT2B | Fwd: CTGACCTGATGCAGACGCAAG  Rvs: AGGAGCCACCTGTAGCTCTCATGTA |
| BMP4 | Fwd: GCCCGGAAGCTAGGTGAGT  Rvs: CAGGAATCATGGTGTCTTGACAGA |
| MYH11 | Fwd: AAACAGGGGATGGACGCAG  Rvs: TGGTGGCATTCATATGGGCG |
| GLI1 | Fwd: GTGCAAGTCAAGCCAGAACA  Rvs: ATAGGGGCCTGACTGGAGAT |
| ACTA2 | Fwd: GCCAAGCACTGTCAGGAATC  Rvs: TTGTCACACACCAAGGCAGT |
| COL1A1 | Fwd: GATTCCCTGGACCTAAAGGTGC  Rvs: AGCCTCTCCATCTTTGCCAGCA |
| FN1 | Fwd: GGGCAACTCTGTCAACGAAG  Rvs: GAGACATGCTTGTTCCTCTGG |
| MMP1 | Fwd: AAGATGAAAGGTGGACCAACAATT  Rvs: CCAAGAGAATGGCCGAGTTC |
| TIMP1 | Fwd: CGG GGC TTC ACC AAG ACC  Rvs: TCA GGC TAT CTG GGA CCG C |
| PBGD | Fwd: GGAGCCATGTCTGGTAACGG  Rvs: CCACGCGAATCACTCTCATCT |

**Suppl. Table 4: Antibodies**

| **Antibody name** | **Manufacturer** | **Dilution** | **Species** |
| --- | --- | --- | --- |
| CDX-2(EPR2764Y) (monoclonal) | ThermoFisher | 1/100 | Rabbit |
| SMA-α (ab5694) (polyclonal) | Abcam | 1/400 | Rabbit |
| E-cadherin (24E10) (monoclonal) | Cell Signalling | 1/200 | Rabbit |
| Villin (SP145) (monoclonal) | Abcam | 1/200 | Rabbit |
| Chromogranin A (ab45179) (polyclonal) | Abcam | 1/500 | Rabbit |
| Vimentin (D21H3) (monoclonal) | Cell Signalling | 1/200 | Rabbit |
| Nanog(D73G4) (monoclonal) | Cell Signalling | 1/100 | Rabbit |
| OCT4 (2750) (monoclonal) | Cell Signalling | 1/100 | Rabbit |
| MUC2 (EPR6145) (monoclonal) | Abcam | 1/400 | Rabbit |
| SOX6 (sc-393314) (monoclonal) | Santa-Cruz Biotechnology | 1/100 | Mouse |
| GLI1 (JF09-08) (monoclonal) | ThermoFisher | 1/100 | Rabbit |
| PDGFRB (G.290.3) (monoclonal) | Invitrogen | 1/100 | Rabbit |
| Human-specific mitochondria (ab92824) (monoclonal) | Abcam | 1/1000 | Mouse |
| Anti-Rabbit IgG (H+L), AF488 | Invitrogen | 1/100 | Chicken |
| Anti-Rabbit IgG (H+L), AF568 | Invitrogen | 1/100 | Donkey |
| Anti-Mouse IgG (H+L), AF594 | Invitrogen | 1/100 | Goat |

*CD31, desmin and Ki67 performed by Nottingham University Hospitals NHS Trust Cellular Pathology Department, datasheets and SOPs available upon request.
