# Supplementary material for: Generation of biologically responsive colon-like intestinal tissue patches from human induced pluripotent stem cells using a rapid co-differentiation platform
Source: Stem Cell Res Ther. 2026 Apr 9;17:182. doi: 10.1186/s13287-026-05006-4 (PMC13173943; doi:10.1186/s13287-026-05006-4)
Supplement: Supplementary file 2 — Supplementary Material 2. [file 13287_2026_5006_MOESM2_ESM.docx]

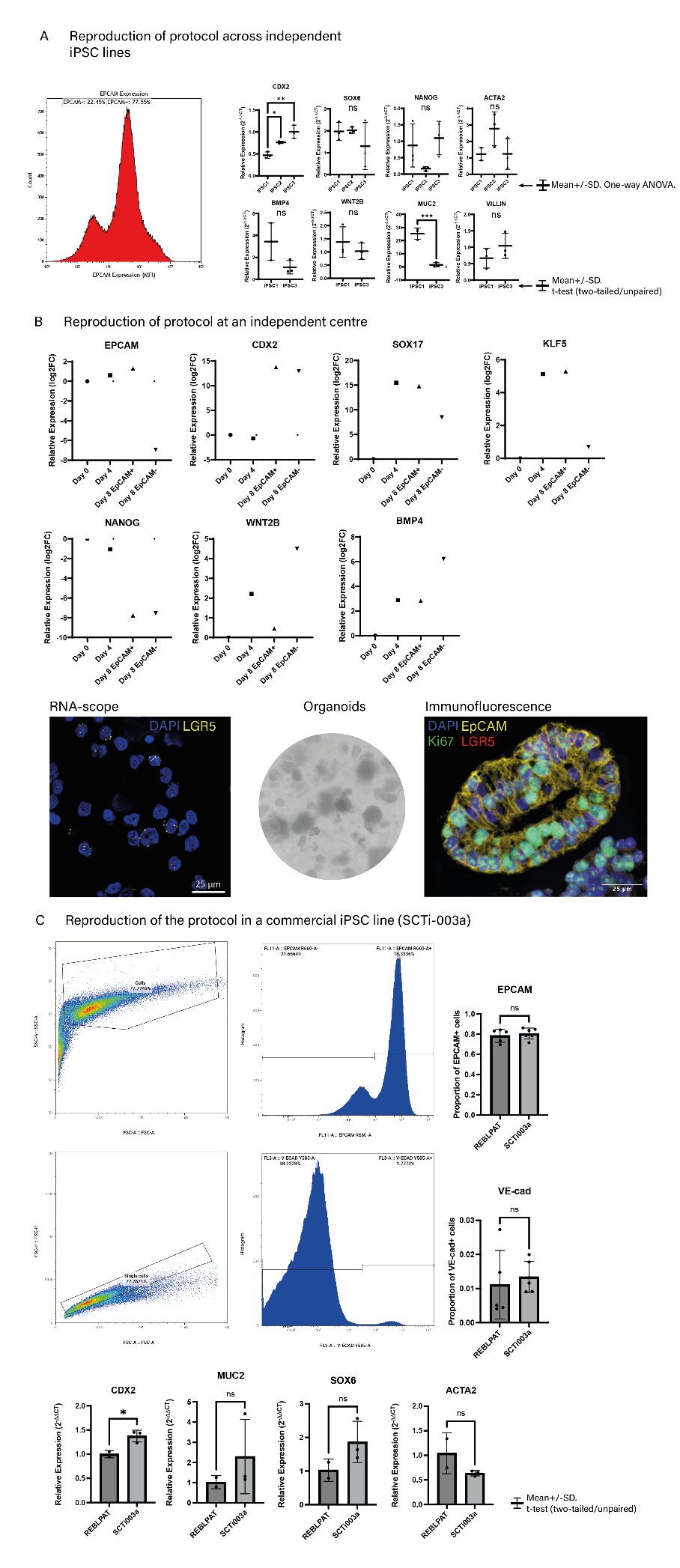


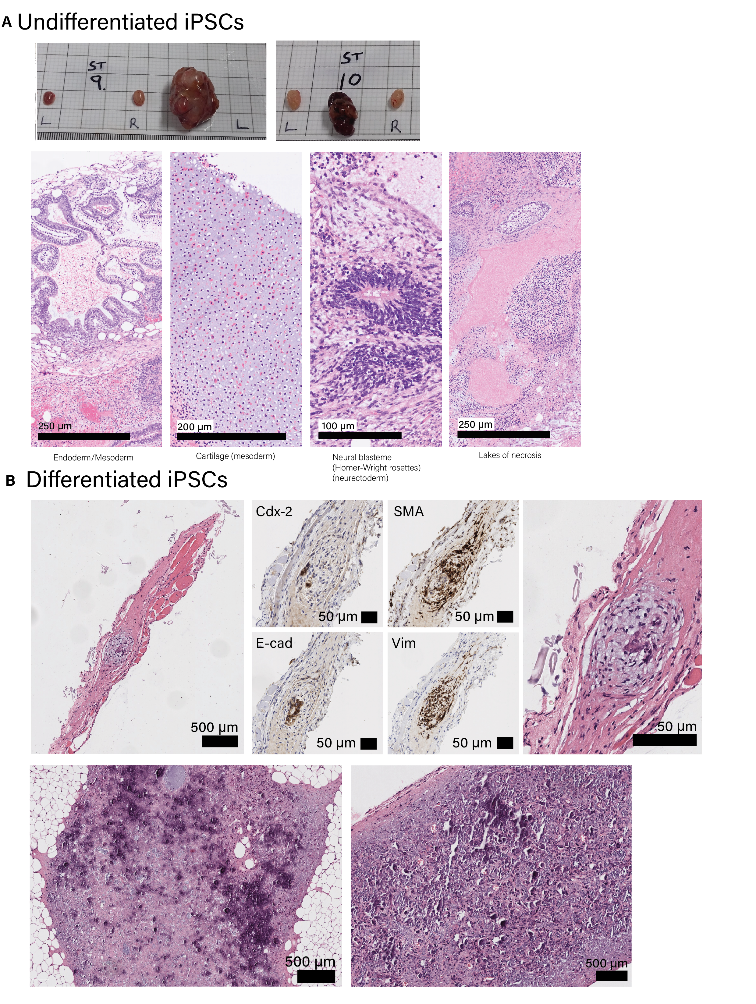


**Figure S1: The differentiation protocol shows consistency across different hiPSCs and laboratories**

1. The co-differentiation (CHACT) protocol was applied to three different hiPSC lines 1-3 derived from both males (REBL-PAT; n=1) and females (BE-31, BE-32; n=2) demonstrated consistency of intestinal epithelial and mesenchymal co-differentiation across multiple hiPSC lines. Differentiated populations consisted of both EpCAM+ and EpCAM- cells (representative flow cytometry histogram from one alternative iPSC line). Evidence of intestinal epithelial (*CDX2, VIL1, MUC2*) and mesenchymal co-differentiation (*ACTA2, WNT2B, SOX6, BMP4*) was demonstrated by qRT-PCR (n=3, one-way ANOVA) as well as suppression of *NANOG* (comparison with hiPSC3 – REBLPAT hiPSCs). Central bar indicates mean and error bars indicate standard deviation. Ns = not significant.
2. The co-differentiation protocol was applied to a fourth hiPSC line (OSTI GI4380) by collaborators at an independent research facility (SM, SI, IT) and showed evidence of intestinal epithelial and mesenchymal co-differentiation. Evidence of intestinal epithelial (*EPCAM, CDX2, SOX17, KLF5*) and mesenchymal (*WNT2B, BMP4*) co-differentiation was shown as well as suppression of pluripotency (*NANOG*) (n=1). EpCAM+ cells were evaluated for human *LGR5* mRNA by RNA-scope (25% across 3 independent replicates) and could form organoids with a mixture of cell types (EpCAM – yellow, Ki67 – green, LGR5 – red, DAPI – blue), representative of n=6. After MACS separation, EpCAM^+^ cells were fixed on a coated slide using cytospin and stained with RNAScope probe for Human *LGR5* mRNA. 7500 cells from 3 independent replicates were counted; 25% of EpCAM+ were positive for *LGR5*, while no cells were positive for LGR5 at day 0.
3. The co-differentiation (CHACT) protocol was applied to a commercial hiPSC line (SCTi-003a) and compared with the original REBL-PAT line. Epithelial and endothelial differentiation capacity was tested by flow cytometry for EPCAM (epithelial) and VE-cadherin (VE-cad; endothelial). No significant difference in the proportions of these two lineages was observed (n=5 REBL-PAT / n=6 SCTi-003a; Student’s t-test). RNA lysates from these conditions were further tested for *CDX-2, MUC2, SOX6* and *ACTA2* expression (n=2 REBLPAT, n=3 SCTi-003a, Student’s t-test). A slight increase in *CDX-2* was seen in SCTi-003a compared with REBLPAT (1.3x higher, p < 0.05) but otherwise gene expression was similar in both cell lines.

**Figure S2: In vivo teratoma assay demonstrates no residual pluripotency in hiPSC-derived intestinal cells using the CHACT protocol**

1. Undifferentiated hiPSCs (positive control): macroscopic photographs of tumours around the testicular subcapsule grown in immunosuppressed (Rag2^-/-^ IL2RG^-/-^) mice following implantation with undifferentiated hiPSCs (n=12). Representative photomicrographs (of twelve images) of H&E stained sections demonstrating features of tissues derived from all three germ layers. From left to right: endodermal / glandular tissue; cartilage (mesoderm); neural blastema / Homer-Wright rosette (ectoderm); lakes of necrosis. Scale bars represent distances indicated in individual photomicrographs.
2. Differentiated hiPSCs (after CHACT protocol) (n=12). All post-mortem tissues were examined by histology. Representative photomicrographs from each specimen where any possible engrafted tissues are shown. Tissue sections were stained with CDX-2, E-cadherin, SMA and vimentin of viable hiPSC-derived intestinal cells. Scale bars represent distances indicated in individual photomicrographs.
